# Beyond causes of death: The social determinants of mortality among children aged 1-59 months in Nigeria from 2009 to 2013

**DOI:** 10.1371/journal.pone.0177025

**Published:** 2017-05-31

**Authors:** Alain K. Koffi, Henry D. Kalter, Ezenwa N. Loveth, John Quinley, Joseph Monehin, Robert E. Black

**Affiliations:** 1 Institute for International Programs/ Department of International Health, Johns Hopkins Bloomberg School of Public Health, Baltimore, Maryland, United States of America; 2 National Population Commission, Abuja, Nigeria; 3 UNICEF, New York, New York, United States of America; 4 USAID, Abuja, Nigeria; Centre Hospitalier Universitaire Vaudois, FRANCE

## Abstract

**Background:**

Millions of children worldwide suffer and die from conditions for which effective interventions exist. While there is ample evidence regarding these diseases, there is a dearth of information on the social factors associated with child mortality.

**Methods:**

The 2014 Verbal and Social Autopsy Study was conducted based on a nationally representative sample of 3,254 deaths that occurred in children under the age of five and were reported on the birth history component of the 2013 Nigerian Demographic and Health Survey. We conducted a descriptive analysis of the preventive and curative care sought and obtained for the 2,057 children aged 1–59 months who died in Nigeria and performed regional (North vs. South) comparisons.

**Results:**

A total of 1,616 children died in the northern region, while 441 children died in the South. The majority (72.5%) of deceased children in the northern region were born to mothers who had no education, married at a young age, and lived in the poorest two quintiles of households. When caregivers first noticed that their child was ill, a median of 2 days passed before they sought or attempted to seek healthcare for their children. The proportion of children who reached and departed from their first formal healthcare provider alive was greater in the North (30.6%) than in the South (17.9%) (p<0.001). A total of 548 children were moderately or severely sick at discharge from the first healthcare provider, yet only 3.9%-18.1% were referred to a second healthcare provider. Cost, lack of transportation, and distance from healthcare facilities were the most commonly reported barriers to formal care-seeking behavior.

**Conclusions:**

Maternal, household, and healthcare system factors contributed to child mortality in Nigeria. Information regarding modifiable social factors may be useful in planning intervention programs to promote child survival in Nigeria and other low-income countries in sub-Saharan Africa.

## Introduction

Globally, the under-five mortality rate declined from 91 deaths per 1,000 live births in 1990 to 43 deaths per 1,000 live births in 2015 [1], but the rates of childhood mortality in sub-Saharan Africa have remained the highest in the world. Nigeria, which has an under-five mortality rate of 109 deaths per 1,000 live births, is among the seven countries in the world with a rate of under-five mortality above 100. Nigeria is the most populous country in Africa, with a total population of approximately 170 million people; however, an estimated 750,000 deaths occurred in children under the age of 5 in 2015 [1].

Nigeria also has the largest economy in Africa, but childhood mortality and socioeconomic and sociocultural characteristics vary markedly across the country’s geographic regions [2]. Previous studies conducted in Nigeria have shown that the northern and southern regions of the country differ in terms of some socioeconomic characteristics [2]. Lower education levels, higher rates of polygamous and early marriage, poorer utilization of modern healthcare facilities, higher proportions of rural residence, and greater poverty have been identified in the northern relative to the southern region [3]. Each of these factors has previously been shown to have strong and additive influences on childhood mortality in different countries [4].

One reason some countries, including Nigeria, have made insufficient progress toward achieving the Millennium Development Goal- 4 (MDG-4) for child mortality reduction is the unavailability of reliable data to inform the measurement of progress and initiation of action. For instance, in Nigeria, there is a lack of population-based data on biological causes of death, and most of the available data are limited to small demographic surveillance sites. In addition, most studies of child mortality lack a well-developed conceptual framework for explaining variations in risk and specifying the biomedical pathways through which social factors ultimately contribute to child mortality. The vital registration system in Nigeria is currently unable to provide reliable national estimates of cause-specific child mortality. Ultimately, the country relies on estimates derived based on statistical modeling, which indicate that most child deaths are caused by diseases that are readily preventable or treatable with proven, cost-effective, and quality-delivered interventions. This modeling approach is often inadequate for the monitoring of trends in the short-term and determination of national healthcare system priorities.

The identification of biomedical and social factors that affect the probability of child survival is important in the effort to contextualize the interventions implemented to achieve under-five mortality rates of 25 or fewer deaths per 1,000 live births by 2030 [5] to the Nigerian situation. Previous studies have suggested that while biomedical interventions can bring about short-term change, only policy interventions at the community and societal levels can have lasting effects [5]. A multitude of factors may be responsible for the missed opportunities that impede the achievement of major improvements in population health.

In the current study, we analyzed social autopsy data derived from the 2014 Verbal/Social Autopsy (VASA) survey in Nigeria. The goal of this study was to provide insights into some of the variables affecting mortality in Nigeria by assessing the prevalence of household, community, and healthcare system factors associated with child mortality from 2009 to 2013. The results of this study may provide additional information regarding modifiable factors that may be useful when planning intervention programs to promote child survival in Nigeria and other low-income countries in sub-Saharan Africa.

## Materials and methods

### Study sites and sample

The data for this study were obtained from the VASA survey, which was undertaken by the National Population Commission (NPC) from February to December 2014. This was the fourth country that conducted a VASA study using technical assistance from the Maternal and Child Epidemiology Estimation (MCEE) (formerly known as the Child Health Epidemiology Reference Group or CHERG), and this study built upon the experiences had by Malawi, Niger, and Cameroon when conducting smaller VASAs. The study protocol and other methodological considerations have been previously described in detail [6].

In Nigeria, the VASA interviews were conducted with women in the 38,522 households in which deaths were identified using 2013 Nigerian Demographic and Health Survey (NDHS) data [2]. All deaths that occurred during the prior 10 years among the children of surveyed women aged 15–49 years were identified via the NDHS. To limit issues related to faulty recall while obtaining an adequate sample size, the VASA study examined deaths among children aged up to 59 months within a 5-year recall period. A total of 1,206 neonatal (0–27 days old) deaths and 2,779 child (1–59 months old) deaths were identified from 2009 to 2013. However, to minimize the burden placed on respondents and because deceased children within the same household were likely to be similar in social and healthcare system factors, the VASA interview focused on one neonatal or child death selected at random within each participating household. This strategy allowed for the inclusion of 986 neonatal and 2,268 child deaths (for a total of 3,254 deaths). Based on an expected proportion of 0.50, these samples sizes enabled confidence intervals of ±0.03 and ±0.02 to be established around the point estimates for the most common causes of neonatal and young child mortality, respectively. Of the 3,254 households sampled, 2,944 (90.5%) had completed the VASA interview; of these cases of mortality, 164 were stillbirths, 723 were neonatal (0–27 days) deaths, and 2,057 were child (1–59 months) deaths. Of the 310 uncompleted interviews, 185 (59.7%) were in households in the northern regions that were not visited because of safety concerns. Restricted working hours, not receiving clearance to enter the clusters on a regular basis, and potential threats were some of the challenges faced by the study teams. The current analysis included only the 2,057 child deaths.

### Data collection tools and the VASA interview

The VASA questionnaire is a combination of the Population Health Metrics Research Consortium (PHMRC) verbal autopsy questionnaire (focused on the biomedical cause of death) and the CHERG Pathway Analysis social autopsy (SA) questionnaire [7] (focused on the well-child and illness events leading up to a death). The CHERG SA questionnaire is a version of an earlier Pathway Analysis instrument [8] that has been updated to more fully elucidate the household, community, and healthcare system factors associated with neonatal and child mortality, including the maternal, preventive, and curative care provided in the event of a neonatal death or stillbirth.

The questionnaire was translated into the following three major languages spoken by most persons in the study area: Yoruba, Igbo, and Hausa. Three local anthropologists were recruited to perform the translations, and another team of experienced staff at the NPC independently back-translated and compared the translations to the original English questionnaire. Discrepancies were then scrutinized to determine the source of the errors and corrected via consultations between the anthropologists and the back translators. An English version of the VASA questionnaire is available in S1 File.

Finally, the translations were inserted into the CSPro software application that was developed to enable direct, field-based Computer Aided Personal Interview (CAPI) capture of the VASA interview data on a netbook computer.

The technical team members who were trained during the pretest/training of trainers (TOT) phase of the project received English interview training and training in the three languages. The trainings were conducted following best practice training procedures, including classroom lectures and presentations, daily reviews, mock interviews, class exercises, and a written test at the end of every module. The trainings also included conducting practice field interviews with mothers who had suffered a recent neonatal or child death. Remedial classes were established for those who did not do well on the tests. A special training was conducted for the supervisors, which focused on the work assignments for team members and backup and transfer of completed cases from the field to the IT manager at the NPC headquarters.

Fourteen interviewing teams carried out the VASA fieldwork, with 11 teams placed in the northern and 3 teams placed in the southern region. The difference in the number of teams per region was due to the greater number of cases identified in the North. The teams carried out field exercises in all 36 states of the country and the Federal Capital Territory (FCT). Each team consisted of a supervisor, three to four interviewers (who were predominantly women), and one driver. The fieldwork was initiated November 1, 2014 and continued through December 2014.

Data quality was ensured by technical team/trainers who also functioned as quality controllers. Data quality was also monitored via the field check tables that were generated by the CSPro program on the supervisors’ netbooks as fieldwork progressed. This methodology was advantageous because the technical team/trainers (or field coordinators) could advise field teams of problems detected during fieldwork. Specifically, tables were generated to evaluate various data quality parameters. The technical team/trainers met in Abuja at the end of each monitoring visit to discuss fieldwork issues and then travelled to states where immediate attention was required. Representatives from USAID and NPC’s commissioners and state directors also monitored the fieldwork process.

### Data analysis

The current analysis of preventive and curative care data followed procedures described previously [9] and was guided by the coverage of the following key indicators along the continuum of the preventive child care provided both inside and outside the home: (a) sociodemographic and household factors associated with child mortality, and (b) illness recognition and care-seeking patterns encompassed by the Pathway to Survival model [7, 8, 10]. All examined interventions have been shown to be efficacious and effective in promoting child survival and, thus, included among the interventions examined by the Lives Saved (LiST) tool [10] or recommended by the World Health Organization (WHO); therefore, these interventions should be accessible to all children. A list and the definitions of some of the operational variables used throughout this paper are available in the article written by Koffi et al. [11].

In addition, a scoring system was developed based on the reported feeding behaviors, activity levels and mental status of the children to assess the impact of perceived illness severity at onset on caregivers’ attempts to seek care for their children. Hence, the following three independent categories of illness severity were constructed: normal/mild, moderate, and severe. Each of these categories had a range of possible scores. The details of the method have been described in a prior paper [11], and the definitions are provided in S1 Fig.

The 2013 NDHS, which served as the platform for the VASA study, used a multistage, stratified sampling methodology with strata defined based on state, rurality and cluster. Therefore, we used design-based weighting to ensure the sample was representative of the overall population of Nigerian women. We also used the Taylor linearization method for variance estimation.

Statistical analyses were conducted using the survey commands (*svy*) in STATA 14.0 (StataCorp, College Station, TX) to adjust for the cluster sampling design, weighting, and calculation of standard errors. For regional analyses, we followed the country’s standard geopolitical divisions, which group the 36 states and the FCT into the following six zones: Northwest, Northeast, North-Central, Southeast, Southwest and South-South. These zones comprised the following two regions: the northern region (herein, the North, i.e., Northwest, Northeast, North-central) and the southern region (herein, the South, i.e., Southeast, Southwest and South-South).

### Ethical considerations

In Nigeria, the VASA study was first approved by the National Health Research Ethics Committee of the Federal Ministry of Health (FMOH) and then approved by the Johns Hopkins School of Public Health’s Institutional Review Board. All respondents provided informed consent before the interview was conducted.

## Results

Interviews were completed for 2,057 (90.7%) of the 2,268 child deaths included in the study sample. The majority of the respondents were the mothers of the deceased children (94.6%).

### Demographic and household characteristics

Table 1 shows the sociodemographic characteristics of the deceased children overall and in the northern and southern regions. Significant associations were identified between several of the sociodemographic variables and childhood mortality and region (all p<0.001 in chi square tests). The median age at illness onset was 11 months in the South and 12 months in the North (median of 12 months overall). More specifically, the majority of the deaths in the South (46.5%) occurred during the post-neonatal period (1–11 months of age), with 22.4% and 24.1% of deaths occurring in the 1–5 month and 6–11 month age groups, respectively. In the North, the proportion of deaths increased incrementally from 14.1% in the 1–5 month age group to 42.7% in the 24–59 month age group. The duration of fatal illness was shorter in the southern region (median = 5 days) than in the northern region (median = 7 days). The gender distributions of the deceased children were similar between the two regions, with 51% boys and 49% girls identified in both the North and the South. Many of the deceased children in the South (66.9%) were born at a healthcare facility, while only 19.1% of the children born in the North had a facility-based birth. More than three quarters (77.6%) of children in the North and more than half (54.2%) of children in the South died at home.

**Table 1:** Characteristics of 2057 deceased children.

Characteristics	Total (N = 2057)	Northern regions (N = 1616)	Southern regions (N = 441)	X^2^ (P-Value)
Median age at illness onset (in months)	12 [n = 2,055; IQR = 7–24 mos*]	12 [n = 1614; IQR = 8–24 mos]	11[n = 441; IQR = 6–12 mos]	33.9 (0.000)
Median illness duration (in days)	6 [n = 2032; IQR = 3–14 days]	7 [n = 1593; IQR = 3–14 days]	5 [n = 439; IQR = 2–9 days]	20.6 (0.000)
Median age death (in months)	12[n = 2057; IQR = 8–24 mos**]	15**[n = 1616; IQR = 9–24 mos]	12 [n = 441; IQR = 6–18 mos]	45.8 (0.000)
Age distribution at death (in months)				
1–5	327 (15.9%)	228 (14.1%)	99 (22.4%)	17.9 (0.000)
6–11	371 (18.0%)	264 (16.4%)	106 (24.1%)	14.14 (0.001)
12–23	568 (27.6%)	434 (26.8%)	135 (30.5%)	2.3 (0.222)
24–59	791 (38.5%)	690 (42.7%)	101 (23.0%)	57.2 (0.000)
Sex				
Boy	1051 (51.1%)	826 (51.2%)	224 (50.8%)	0.0 (0.913)
Girl	1006 (48.9%)	789 (48.9%)	217 (49.2%)	
Place of birth				
Hospital	375 (18.2%)	201 (12.4%)	174 (39.5%)	779.6 (0.000)
Other health provider or facility	162 (7.9%)	41 (2.5%)	121 (27.4%)	
On route to a health provider or facility	7 (0.3%)	7(0.4%)	0 (0.0%)	
Home	1458 (70.9%)	1364 (84.4%)	95 (21.4%)	
Other	55 (2.7%)	3 (0.2%)	52 (11.7%)	
Place of death				
Hospital	369 (18.0%)	261 (16.2%)	108 (24.5%)	134.1 (0.000)
Other health provider or facility	76 (3.7%)	47 (2.9%)	30 (6.7%)	
On route to a health provider or facility	70 (3.4%)	31 (1.9%)	40 (9.0%)	
Home	1493 (72.6%)	1254 (77.6%)	239 (54.2%)	
Other	45 (2.2%)	21 (1.3%)	25 (5.6%)	
Don’t know	3 (0.1%)	3 (0.2%)	0 (0.0%)	

Notes:

* mos: months;

**The variable ‘Age at death’ was either calculated by the CSPro program or stated by the respondent. It was recorded either in months or years for 1–59 month deaths. 55.4% of the children had their age recorded in years in the north (as opposed to 44% in the south). Age at death is expressed in months, hence the discrepancy between (age at illness onset + illness duration) and age at death, especially in the north.

Table 2 shows that almost all the evaluated characteristics of the mother, her domestic partner, and the household were significantly associated with region (p<0.001). More than two-thirds (71.5%) of the mothers of deceased children married early in adolescence (<16 years) in the North, whereas only 5.9% of mothers married early in the South. Many the mothers in both regions were 25 years or older when their index child died (57.2% in the North and 76.9% in the South). Women who had no formal schooling constituted a higher percentage (72.5%) of the mothers in the North than in the South (11.1%). The majority (47.2%) of the mothers in the South had some secondary education. The data revealed a similar pattern in the association between paternal education and region. The majority of the deceased children in the northern region resided in the poorest households in the country. Conversely, 80.5% of the deceased children in the South lived in middle-income and richer households. More than three-fourths (76.4%) of the deceased children lived in households located in rural areas. The urban/rural distribution varies greatly by region; the majority of households (86.3%) in which a deceased child resided in the North were located in the rural areas, while 40.0% of deceased children in the South resided in a rural area. Furthermore, of the 1,395 households in which deceased children resided in rural areas of the north, 83.6% were in the two bottom quintiles of wealth, whereas 23.5% of the 176 rural households in which deceased children resided in the South were in the bottom two wealth quintiles. Among households that experienced a child death, the median household sizes were 7 and 5 members in the North and South, respectively. The median time required to travel from the caregivers’ household to their usual healthcare center was 30.0 minutes in both the North and South.

**Table 2:** Characteristics of the mother and her household, 2057 child deaths.

Characteristics	Total (N = 2,057)	Northern regions (N = 1,616)	Southern regions (N = 441)	X^2^ (P-Value)
**Maternal Characteristics**				
Married or living with a man	1975 (96.1%)	1571 (97.2%)	405 (91.8%)	27.4 (0.000)
Mother’s Age when first married (Median in years)	15 [n = 1942; IQR* = 14–18 years]	15 [n = 1543; IQR = 12–16 years]	20 [n = 399; IQR = 18–24 years]	662.7 (0.000)
12–15 years	1147 (58.5%)	1123 (71.5%)	24 (5.9%)	897.8 (0.000)
16–19 years	447 (22.6%)	337 (21.5%)	110 (27.2%)	
20–24 years	252 (12.8%)	71 (4.5%)	181 (44.7%)	
25 or more years	101 (5.1%)	16 (1.0%)	85 (21.1%)	
Don’t know	28 (1.4%)	23 (1.5%)	5 (1.1%)	
Mother’s age at time of child death (Median in years)	27 [n = 2047; IQR = 22–33 years]	26 [n = 1607; IQR = 22–32 years]	30 [n = 440; IQR = 25–35 years]	66.2 (0.001)
< = 19 years	252 (12.2%)	229 (14.2%)	22 (5.0%)	60.2 (0.000)
20–24 years	532 (25.9%)	454 (28.1%)	78 (17.7%)	
> = 25 years	1263 (61.4%)	924 (57.2%)	339 (76.9%)	
Don’t know	10 (0.5%)	8 (0.5%)	2 (0.4%)	
Mother’s median years of maternal schooling	0 [n = 1961; IQR = 0–6 years]	0 [n = 1520; IQR = 0–0 years]	6 [n = 441; IQR = 6–12 years]	685.8 (0.000)
None (0 years)	1220 (59.3%)	1171 (72.5%)	49 (11.1%)	723.8 (0.000)
Primary (1–6 years)	417 (20.3%)	232 (14.4%)	185 (41.9%)	
Secondary or more (7 or more years)	324 (15.7%)	116 (7.2%)	207 (47.0%)	
Don’t know	96 (4.7%)	96 (5.9%)	0 (0.0%)	
Father’ median years of schooling	2 [n = 1907; IQR = 0–9 years]	0 [n = 1491; IQR = 0–6 years]	7 [n = 416; IQR = 6–12 years]	307.5 (0.001)
None (0 years)	942 (45.8%)	908 (56.2%)	33 (7.5%)	371.7 (0.000)
Primary (1–6 years)	433 (21.0%)	258 (16.0%)	175 (39.6%)	
Secondary or more (7 or more years)	533 (25.9%)	325 (20.1%)	208 (47.2%)	
Don’t know	150 (7.3%)	126 (7.7%)	25 (5.7%)	
**Household Characteristics**				
Type of place of residence				
Urban	486 (23.6%)	221 (13.7%)	265 (60.0%)	412.2 (0.000)
Rural	1570 (76.4%)	1394 (86.3%)	176 (40.0%)	
Wealth quintiles**				
Poorest	663 (32.2%)	652 (40.3%)	12 (2.6%)	603.4 (0.000)
Poorer	647 (31.5%)	573 (35.4%)	75 (16.9%)	
Middle	349 (17.0%)	233 (14.4%)	116 (26.3%)	
Richer	254 (12.4%)	123 (7.6%)	131 (29.6%)	
Richest	144 (7.0%)	36 (2.2%)	108 (24.6%)	
Household size (Median)	6 [n = 2056; IQR = 4–8]	7 [n = 1615; IQR = 5–9]	5 [n = 441; IQR = 4–6]	94.4 (0.000)
Median travel time to usual health facility (min)	30 [n = 2037; IQR = 20–60 min]	30 [n = 1598; IQR = 20–60 min]	30 [n = 439; IQR = 15–60 min]	1.1 (0.291)
Median time at current residence (in years)	12 [n = 2012; IQR = 7–20 years]	14 [n = 1574; IQR = 8–22 years]	8 [n = 438; IQR = 5–14 years]	114.3 (0.000)

Notes:

*IQR: Interquartile Range;

**This variable comes from the 2013 NDHS data.

### Preventive home care

**Fig 1:**
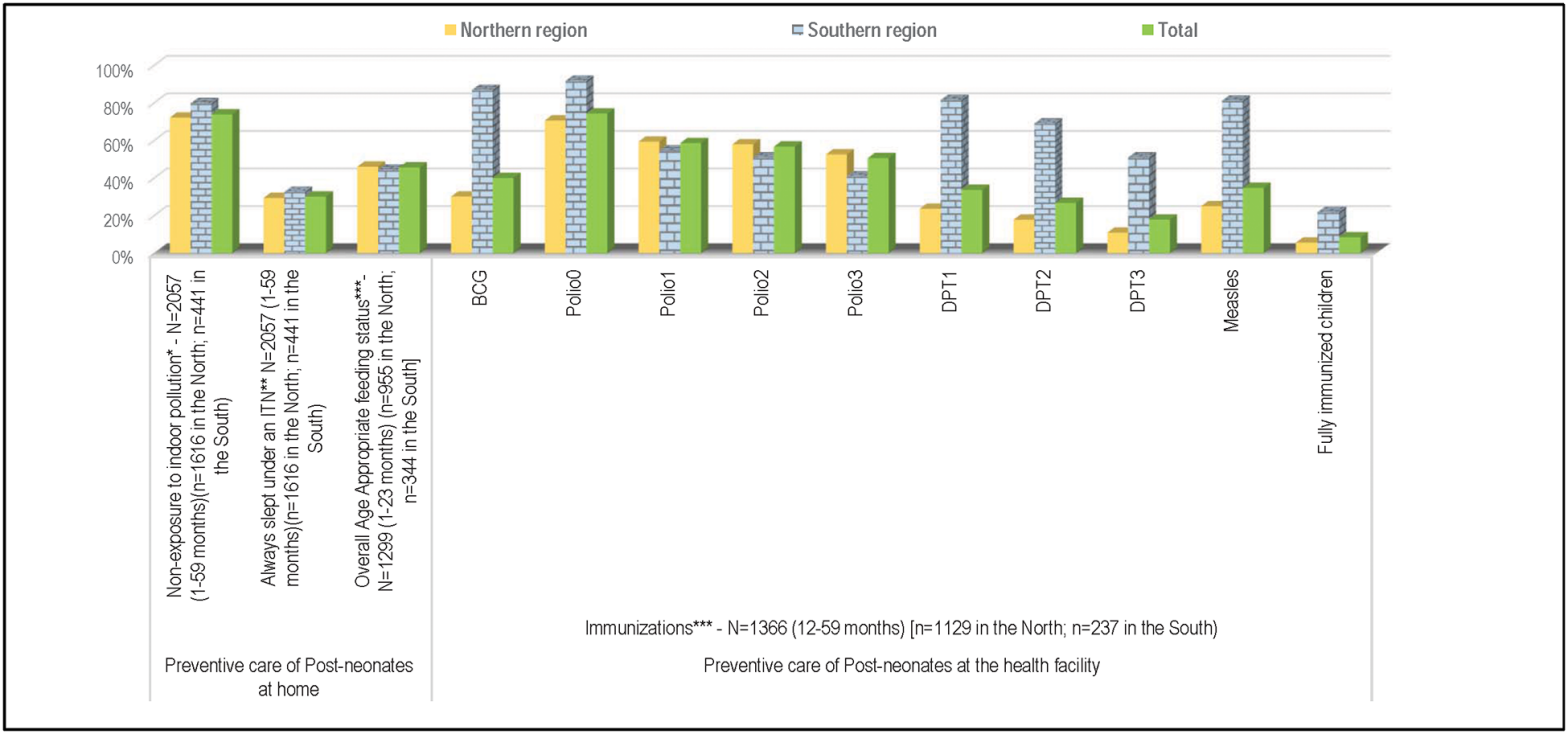
Coverage along the continuum of care for young children in Nigeria, from 2009–2013. Notes: *: Proportion of children who were NOT usually beside or carried by their mother when she cooked inside the home. **: Insecticide-treated bed net. ***: Children whose fatal illness started at 0–23 months and satisfied either of the following conditions: (i) Child’s illness began before 6 months of age (0–5 months), he/she was being breastfed at the time of fatal illness and was not given anything but breast; (ii) Breastfed children whose fatal illness started at 6–8 months old and 9–23 months old who received, respectively, at least two and three complementary non-liquid feedings each day; (iii) Child's fatal illness started at 6–23 months old and he/she received at least four replacement feeds each day (including milk and solid, semi-solid and soft foods) milk as food. ****Information on immunizations was obtained either from the vaccination card or when there was no written record, from the respondent (mainly the mother). Polio0 is the Polio vaccination given at birth; Fully Immunized children received BCG, measles, and three doses each of DPT/PENTA and polio vaccine (excluding polio vaccine given at birth).

Fig 1 displays the various types of preventive home care received by the deceased children prior to his/her fatal illness. Approximately three in four children (73.8%) were not likely to be exposed to smoke (i.e., he/she was usually away from the mother when she cooked inside the house), with a significantly higher proportion of children reported to avoid smoke exposure in the South than in the North (79.8% versus 72.2%, χ2 (1) = 10.4, p = 0.033). Less than one third of the children always slept under an insecticide-treated bed net before their fatal illness began, and no significant regional difference was identified. Overall, among children in whom illness was first identified at 0–23 months (n = 1,299), 46% were being fed appropriately at the time their fatal illness began, and no difference was observed between the North (46.0%) and the South (44.0%). In both the northern and the southern regions, the proportions of children whose illness symptoms first appeared at 0–23 months and were exclusively breastfed decreased incrementally by age group at illness onset (0–5 months: North 26.0%, South 33.8%; 6–11 months: North 11.4%, South 7.0%; 12–23 months: North 4.7%, South 0.1%). Fig 1 shows the percentage of deceased children aged 12–59 months (n = 1,359) who had been vaccinated against each of the six major preventable childhood diseases by the age of one year. Overall, only 8.0% of the children were fully immunized against these diseases before they reached their first birthday, and a significant difference was observed between the immunization rate in the northern (6.0%) and southern (22.0%) region (χ2 (1) = 67.1, p<0.001). The polio vaccine had the highest rates of coverage in the North (ranging from 53% to 59% for one to three doses), and in the South, BCG, DPT or PENTA, and measles demonstrated the highest rates of coverage, ranging from 51% to 87%. Deceased children in the North were unlikely to be fully immunized against DPT or PENTA by the age of one year (only 11% had received all three doses).

### Curative care seeking

**Fig 2:**
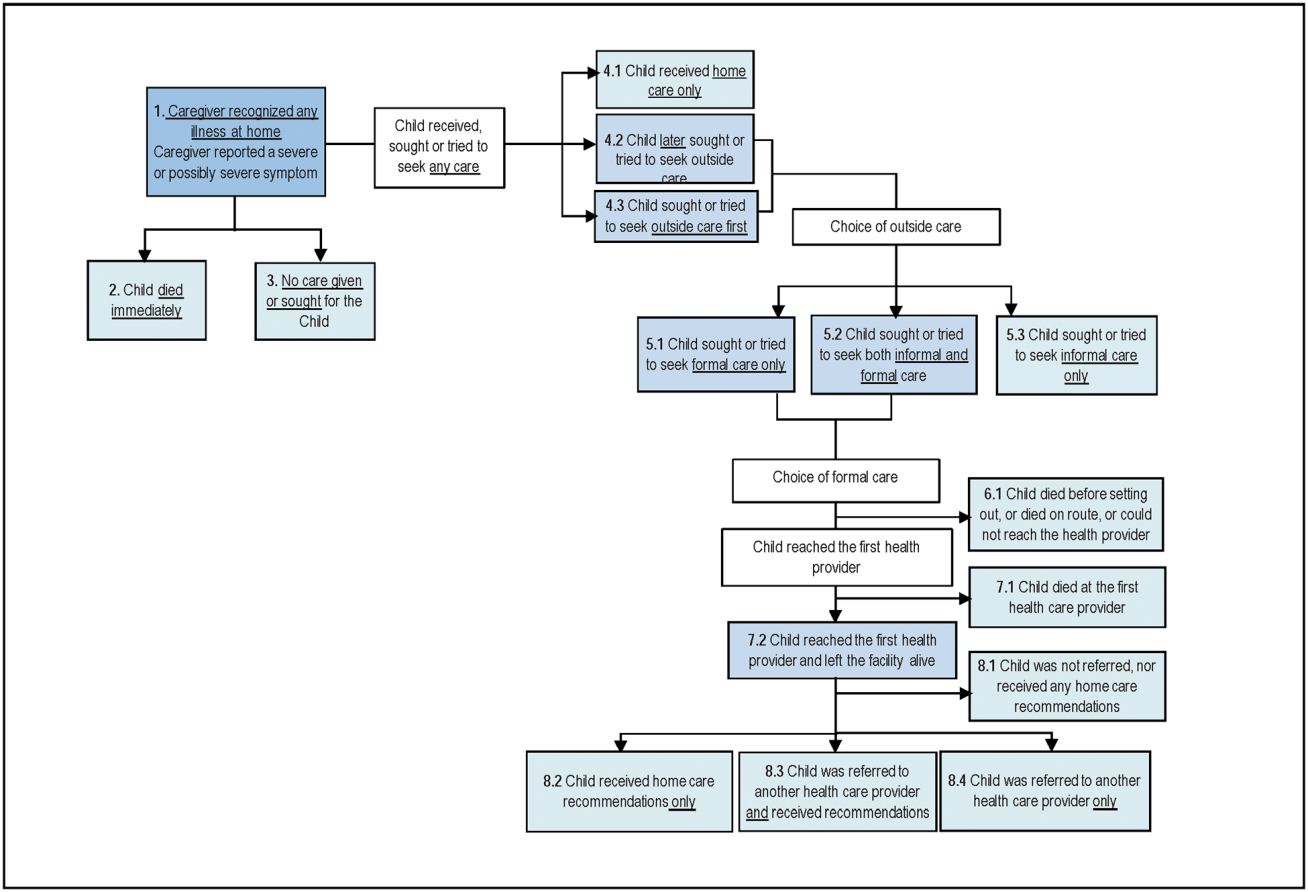
The “Pathway to Survival” component and indicators.

Fig 2 exhibits the steps and possible breakdowns in the Pathway to Survival that may have contributed to mortality in the included children. Table 3 depicts the distribution of these indicators by region and the results of chi-square tests for their corresponding associations. These analyses included only the children whose caretakers provided information on the types of curative actions taken for the illness (i.e., n = 1,615 in the North and n = 441 in the South). When the caregivers of these 2,056 children first noticed that the child was ill, nearly all (97.1%) of them recognized the presence of severe or possibly severe signs or symptoms. Healthcare was obtained or sought outside the home for almost all (84.6%, n = 1,739) the children; 63.9% of the caregivers of deceased children in the North first sought or attempted to seek outside care, while only 51.5% did so in the South (p<0.001). A significantly greater proportion of caregivers in the South (38.1% vs. only 19.4% in the North; p<0.001) first sought healthcare inside the home and later sought or attempted to seek formal healthcare. In total, more than half (53.9%) of the children in the South and 44.5% of children in the North received or had caregivers who attempted to seek formal care only; additionally, another 20.4% and 15.6% of children received or had caregivers who attempted to seek both informal and formal care in the South and the North, respectively. In other words, 74.3% of children in the South and 60.1% of children in the North received or had caregivers who attempted to seek formal care (χ2 (1) = 20.3, p<0.001).

**Table 3:** Pathway to survival component and indicators.

Pathway to Survival Component and Indicators	Total	Northern regions	Southern regions	X^2^ (P-Value)
**Illness recognition at home**				
**1.** Caregiver recognized any illness	2056 (100%)	1615 (100%)	441 (100%)	
Caregiver recognized sign(s) of possibly severe or severe illness	1996 (97.1%)	427 (96.8%)	1569 (97.2%)	0.3 (0.673)
**Care-seeking patterns**				
**2.** Child died immediately	87 (4.2%)	83 (5.2%)	4 (0.8%)	15.9 (0.000)
**3**. No care given or sought for child	91 (4.4%)	75 (4.7%)	16 (3.6%)	0.9 (0.420)
**4.1** Child received home care only	139 (6.7%)	113 (7.0%)	26 (5.9%)	0.6 (0.474)
**4.2** Child sought or tried to seek outside care as first action	1258 (61.2%)	1031 (63.9%)	227 (51.5%)	22.3 (0.000)
**4.3** Child sought or tried to seek outside care as second action	481 (23.4%)	313 (19.4%)	168 (38.1%)	67.7 (0.000)
**Choice of outside care**				
**5.1** Child formal care only	956 (46.8%)	718 (44.5%)	238 (53.9%)	12.5 (0.005)
**5.2** Child informal and formal care	342 (16.7%)	252 (15.6%)	90 (20.4%)	5.6 (0.0372)
**5.3** Child informal care only	441 (21.4%)	374 (23.1%)	67 (15.2%)	12.8 (0.008)
**Choice of any formal care**				
**6.1** Child died before setting out, or died on route, or could not reach the health care provider	396 (19.3%)	242 (15.0%)	154 (35.0%)	88.6 (0.000)
**7.1** Child reached the first health care provider and died at the facility	328 (16.0%)	234 (14.5%)	95 (21.5%)	12.6 (0.008)
**7.2** Child reached the first health provider and left the facility alive	574 (27.9%)	495 (30.6%)	79 (17.9%)	27.8 (0.000)
**Decision of health provider at discharge (of the children who left the health facility alive)**				
**8.1** child was not referred, nor received any home care recommendations	275 (48.0%)	248 (50.3%)	27 (33.7%)	7.7 (0.018)
**8.2** Child received home care recommendations only	230 (40.1%)	195 (39.4%)	36 (44.9%)	0.9 (0.395)
**8.3** Child was referred to another health care provider and received home care recommendations	49 (8.5%)	36 (7.3%)	13 (16.0%)	7.0 (0.012)
**8.4** Child was referred to another health care provider only	19 (3.4%)	15 (3.1%)	4 (5.3%)	1.1 (0.413)

In addition, more than three-quarters (75.5%, 68/90) of both formal and informal healthcare professionals who provided care to the children or from whom caregivers attempted to seek care in the South consisted of pharmacists or drug sellers, while these healthcare professionals only comprised 44.9% of providers reported in the North (113/252), (χ2 (1) = 25.3, p<0.001). Conversely, 45.5% (170/374) and 34.3% (23/67) of caregivers sought only informal care from pharmacists or drug sellers in the North and the South, respectively. Overall, when informal care was sought for the children’s illnesses, more than half (58.0%, 91/157) of caregivers in the South and 45.2% (283/626) of caregivers in the North (χ2 (1) = 7.8, p = 0.016) sought care from pharmacists or drugs sellers.

**Fig 3:**
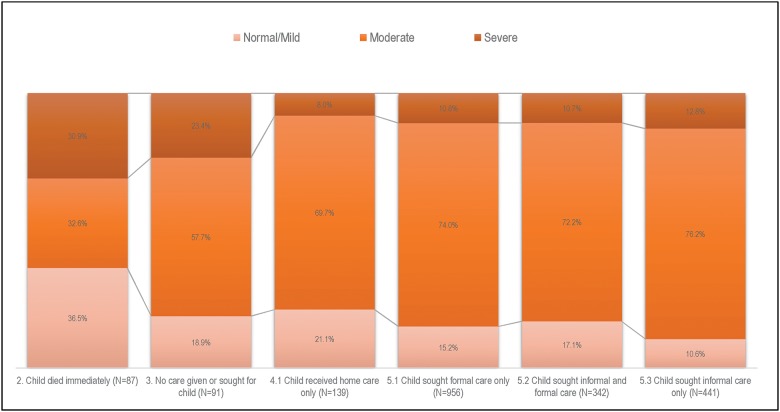
Perceived illness severity at illness onset by care-seeking patterns, Nigeria 2009–2013.

The majority of the children were already moderately to severely ill at illness onset (Fig 3); 81.1% of the children who did not receive any care were moderately to severely ill, and more than three-quarters of children whose caregivers provided only home care were moderately to severely ill at illness onset. Furthermore, 23.4% (21/91) of children who did not receive any care were severely ill, while 11.1% (207/1878) of children who received any care had an illness of similar severity (χ2 (1) = 12.9, p<0.001).

The median duration (delay) from illness onset until seeking formal care was 2 days among children whose caregivers sought or attempted to seek both informal and formal care and children whose caregivers sought or attempted to seek formal care only, and no regional difference was observed (χ2 (1) = 0.0, p = 0.905). Children who first received home care (but whose caregivers later sought or attempted to seek some formal care) experienced a longer delay prior to seeking formal care (median = 3 days, IQR = 2–6 days]) than did children whose caregivers first sought or attempted to seek formal care (median = 2 days, IQR = 1–3 days) (χ2 (1) = 144.8, p<0.001).

**Fig 4:**
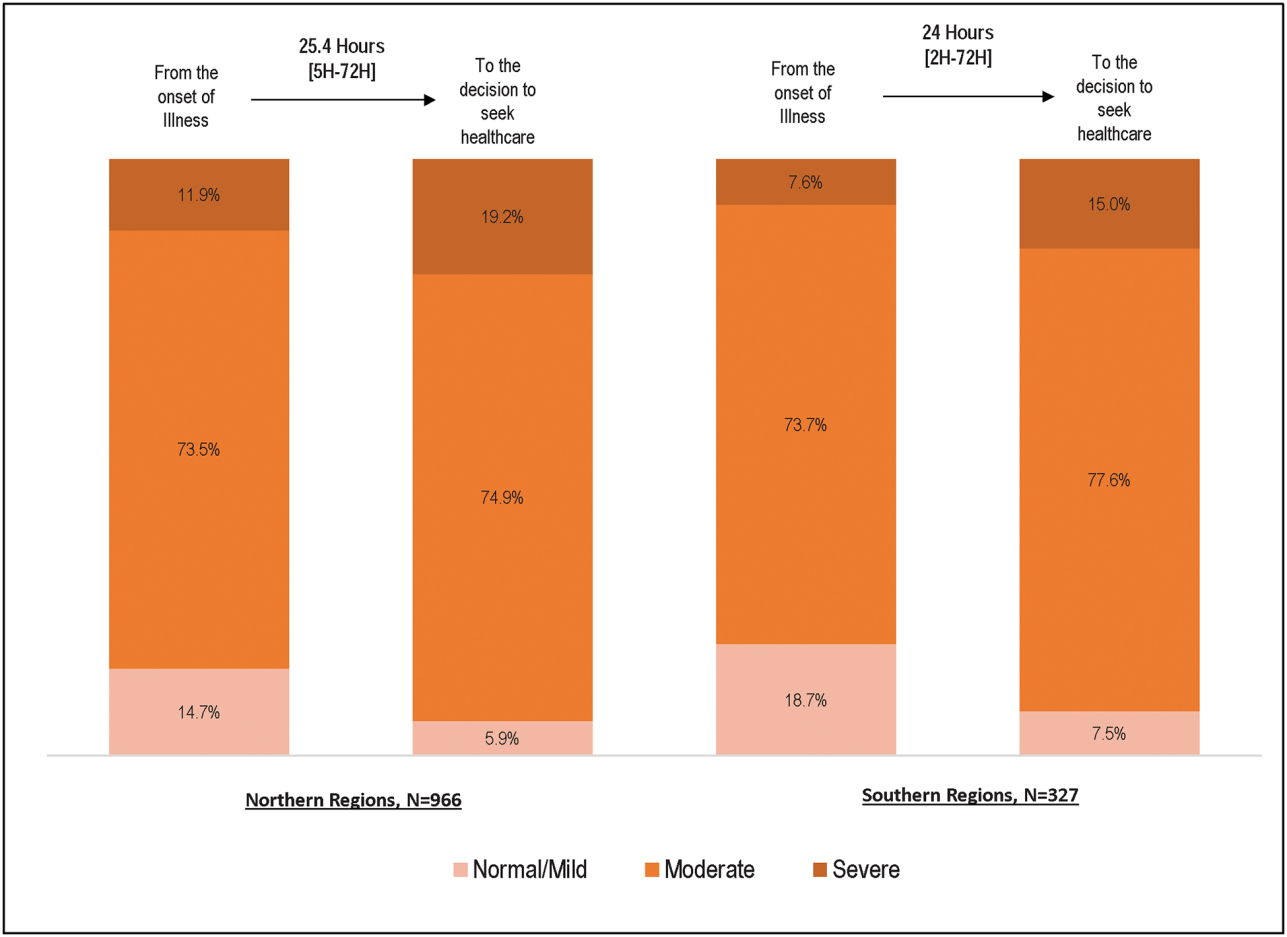
Illness severity ranking at onset and at decision to seek care among children for whom caregivers tried to seek or sought some formal care (N = 1293*), by region, Nigeria 2009–2013. Note: *5 children had missing information that did not allow their illness severity ranking.

However, among those who sought formal care, the decision to do so occurred a median of 25.4 hours in the North and 24.0 hours in the South after illness onset (Fig 4). A median 2-day delay in seeking formal care was observed regardless of the perceived severity of illness (χ2 (2) = 4.1, p = 0.131).

Furthermore, among children whose caregivers sought or attempted to seek formal care (n = 970 and n = 328, excluding the 4 and 1 children with missing perceived illness severity data in the north and south, respectively), the proportion perceived to be severely ill increased from 11.9% at the time of illness onset to 19.2% when the decision was made to seek formal care in the North and from 7.6% to 15.0% in the South (Fig 4).

Although nearly three-quarters (74.3%) of caregivers in the South (versus 60.1% in the north) reported that they attempted to seek some formal care on behalf of their sick children, they were less likely to reach the healthcare facility than were those in the north (χ2 (1) = 88.6, p<0.001); reasons for this decreased likelihood included child death before departure, child death en route, and inability to reach the healthcare provider. The proportion of children who were moderately ill at the time when their caregiver decided to seek care on their behalf and died before reaching their healthcare provider was greater in the South (77.4%; albeit not significantly, χ2 (1) = 4.8, p = 0.079) than in the North (66.6%). On the other hand, the proportion of severely ill children who died before reaching their healthcare provider was higher in the North (24.5%) than in the South (14.7%) but not significantly so (χ2 (1) = 5.0, p = 0.060).

**Fig 5:**
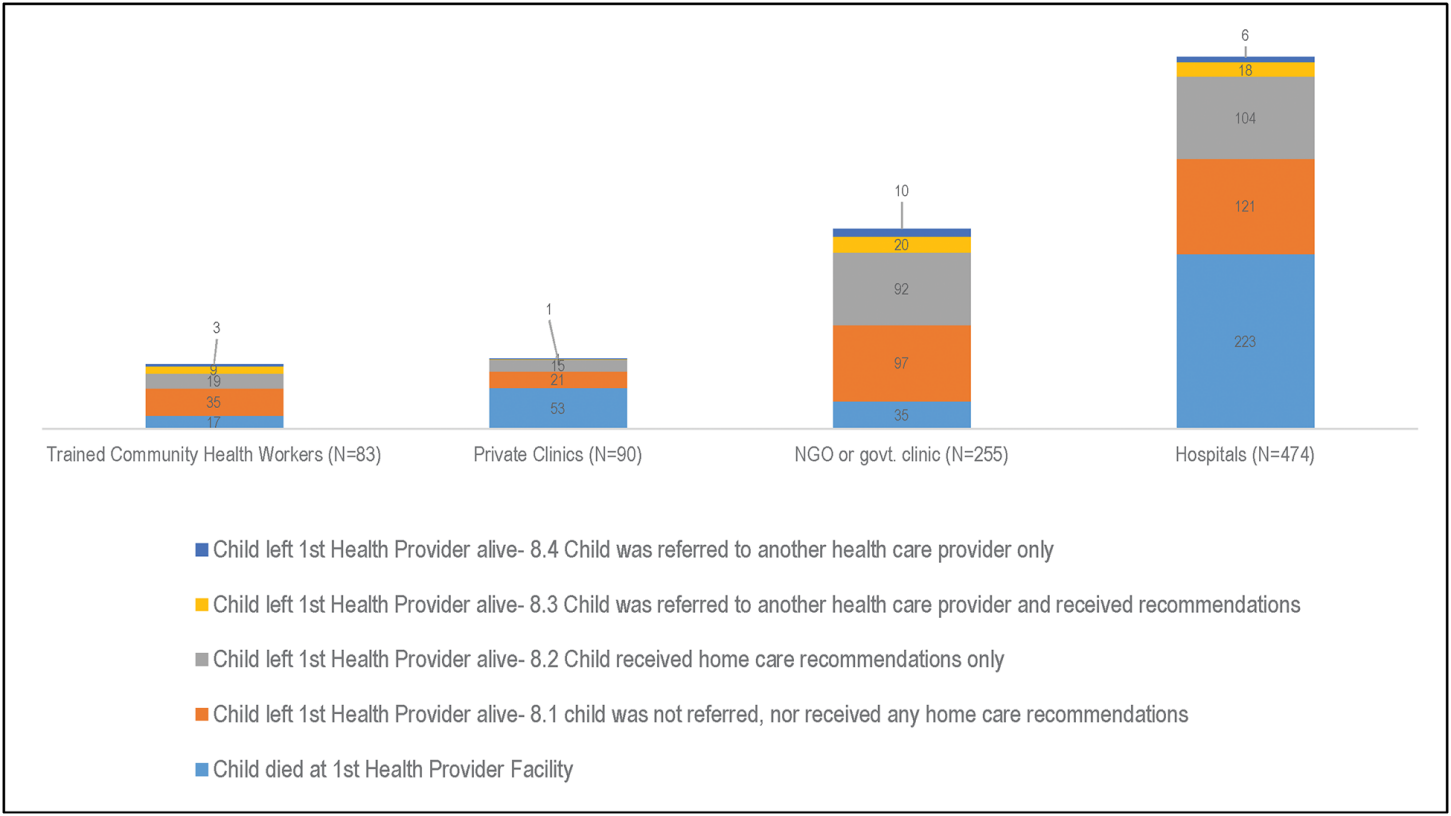
Type of first formal health provider reached by caregivers with their ill children.

Overall, 901 (n = 727 in the North; n = 174 in the South) of the 1,298 children whose caregivers attempted to seek formal care reached a healthcare provider before the child died. The healthcare providers reached first were, in descending order of frequency, hospitals (n = 474), non-governmental (NGO) or governmental (GOV) clinics (n = 255), private clinics (n = 90), and trained community health providers (n = 83) (Fig 5). The distributions of the following first reached healthcare providers differed by region: hospitals (49.7% in the North, 64.4% in the South), NGO or GOV clinics (32.9% in the North, 9.1% in the South), private clinics (9.3% in the North, 13.0% in the South), and trained community health providers (8.1% in the North, 13.5% in the South). The proportion of children who first reached hospitals was significantly higher in the South than in the North (χ2 (1) = 12.5, p = 0.004), but the proportion first reaching NGO or GOV clinics was significantly greater in the North than in the South (χ2 (1) = 40.2, p<0.001). The delay in obtaining formal care seeking did not vary significantly by the type of healthcare provider reached first (χ2(3) = 3.2, p = 0.368).

Nearly all (94.7%) the children were moderately or severely ill when they reached their first healthcare provider. No statistically significant regional difference was identified in perceived illness severity among this subset of children.

More than half (58.9%) of the children who reached a private clinic and almost half (47.0%) of those who reached a hospital died at those health facilities (Fig 5). Only one in four (26.1%) children who reached a private clinic and 18.0% of children who reached a hospital were perceived to be severely ill at the time their caregivers decided to seek care on their behalf.

The proportion of children who reached their first healthcare provider’s facility and died there and were severely sick when their caregivers decided to seek formal care was significantly higher in the North (30.2%) than in the South (18.3%) (χ2 (1) = 5.0, p = 0.046). However, the overall proportion of children who reached their first healthcare provider and died there was significantly higher in the South (21.5%) than in the North (14.5%) (χ2 (1) = 12.6, p = 0.008) (Table 3).

More children in the North (30.6%) than in the South (17.9%) left their first healthcare provider alive (χ2 (1) = 27.8, p<0.001). Of the 574 children who left their first provider alive, 95.6% (n = 548; or n = 477 in the North and n = 71 in the South) were moderately or severely sick at discharge. Only 11.9% (n = 68) of the children were referred to another healthcare provider (some also received home care recommendations), with the following statistically significant difference observed between regions: 10.5% (50/477) in the North and 23.2% in the South (17/71), (χ2 (1) = 9.6, p = 0.007). According to the caregivers, the majority of the children (59 out of 68, or 86.8%) were referred to a second provider because their first provider was not capable of managing the problem, while 24.5%-26.5% believed their child’s condition required supplies (e.g., drugs, IV, oxygen) and/or equipment (e.g., X-ray machine) that were not available at the first facility during the child’s visit, and 3 (4.6%) did not know the reason their child was referred to a second provided (results not shown). Fifty-six (56/68 = 82.5%) sought care from the referred second health provider. More than 40% of the children received only home care recommendations from their first healthcare provider at discharge.

**Fig 6:**
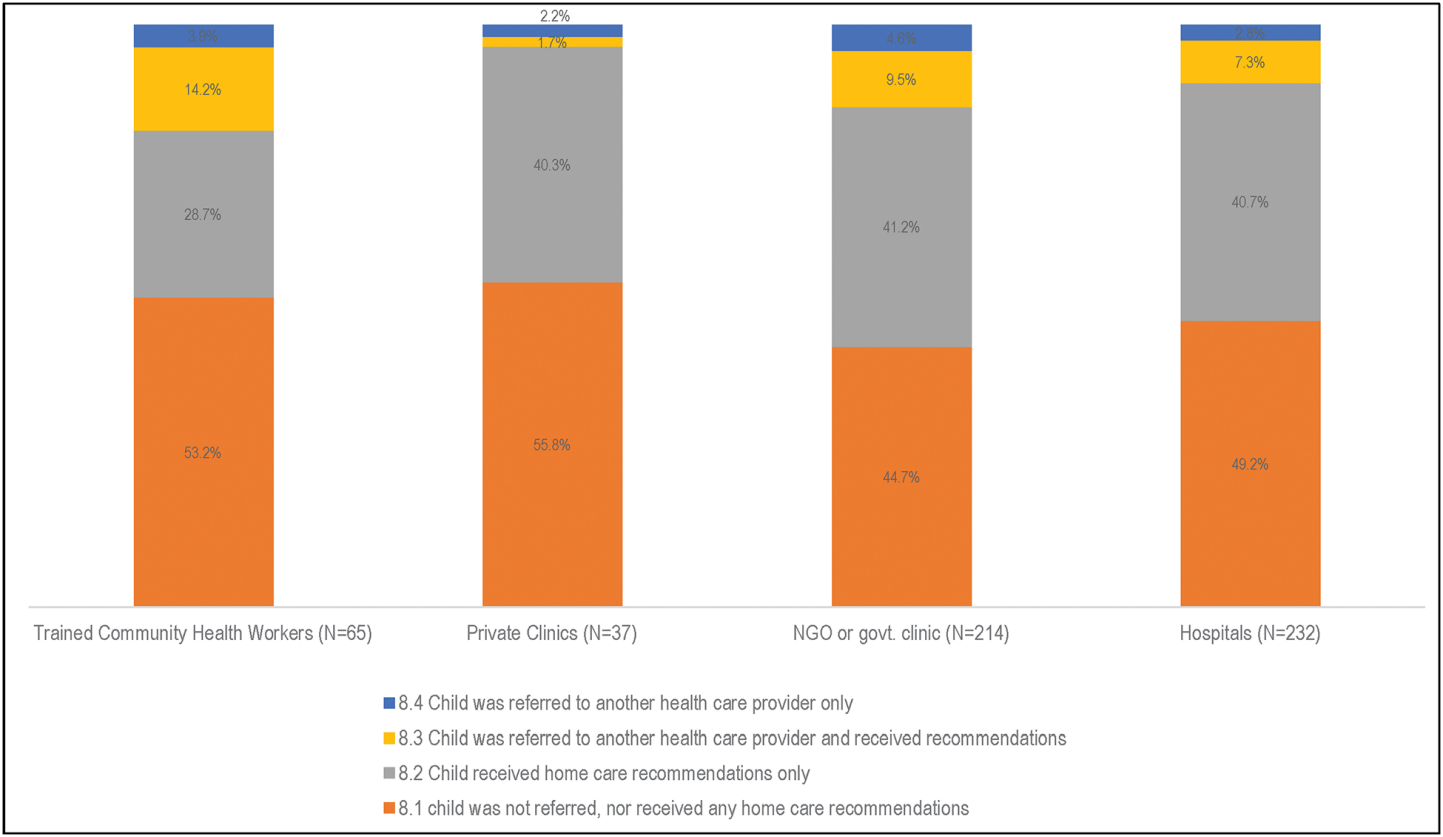
Decision of first healthcare provider for children moderately or severely sick at discharge (N = 548), by health facility/provider.

Fig 6 focuses on the 548 children who had moderate or severe illness at discharge from the first healthcare provider. Trained health workers were most likely to refer these children to another healthcare provides (18.1%), while private clinic facilities were the least likely (3.9%).

Furthermore, 70% (n = 384) of the moderately/severely ill children the not receive additional formal healthcare either because their caregiver did not attempt to reach this provided at all (n = 342) or they tried but did not reach the provider before they died (n = 42). Fourteen percent (n = 77) reached another health provider and left the facility alive, while 16% (n = 87) reached another provided and died at their facility. we estimated how long after leaving the first provider the 342 and 77 children died, and the former died 2 days (median) (IQR: 1–5 days) after leaving the first healthcare provider, and the latter died a median of 8 days after first discharge (median) (IQR: 4–14 days) (χ2 (1) = 86.9, p<0.001).

Similarly, of the 87 children who died at the second health provider, 29 were not admitted to that facility; hence, we assumed they died immediately after reaching the facility. We also added the number of days the other children (n = 58) stayed at the latter health facility to the time elapsed between seeking care from the first and second providers and approximated that these children died a median duration of 4 days (median) (IQR = 2–11 days) after leaving the first provider. The results of the chi-square test indicated that the two medians (4 days versus 2 days) were significantly different (χ2 (1) = 22.7, p<0.001).

### Barriers to seeking healthcare

**Fig 7:**
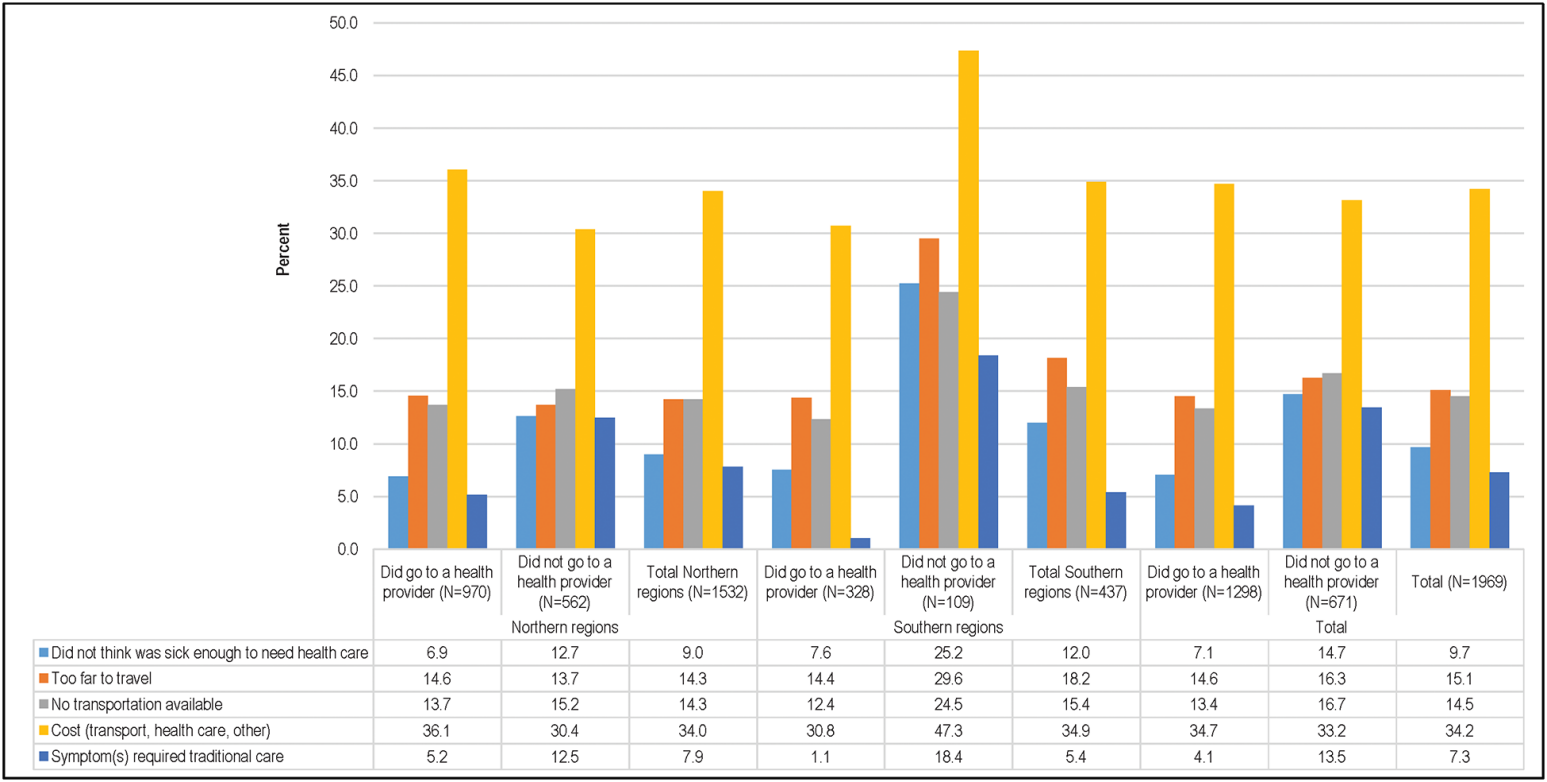
Main care-seeking constraints for child deaths (N = 1969).

Fig 7 shows the barriers to seeking care for fatal illnesses for all children except those who died immediately (n = 87). Caregivers who did not take or attempt to take their children to a formal healthcare provider (n = 671) were asked to describe what prevented them from going to the healthcare provider, while those who did take their child to receive healthcare (n = 1,298) were asked what constraints they had to overcome during this process. In the North, the proportions of caregivers reporting barriers were similar among those whose children did and did not receive care. However, in the South, caregivers who did not take their children to visit a healthcare provider reported these problems more frequently (18%-47%) than those who did (1%-31%). Overall, cost (34.2%), lack of transportation (24.3%), and distance (15.1%) were the most commonly cited constraints to seeking care from a healthcare provider. Cost was an especially common complaint among those in the South who did not seek care for their children (47%). Approximately 15% of respondents whose children were not taken to receive care stated that they did not think that the child was sick enough to seek formal healthcare. This practice was especially common in the South (25.2%).

## Discussion

The aim of this paper was to describe the pathways through which social factors ultimately contributed to child mortality in Nigeria from 2009 to 2013. An important contribution made by this study is the insight into modifiable social factors that will be vital to health policymakers in both government and non—governmental organizations working in Nigeria as they develop new policies and programs and allow for better resource planning in the post-2015 period.

Similar to previous studies in developing countries, our study detected an association between region (North and South Nigeria) and some sociodemographic characteristics of the mother, her domestic partner, and the household. These associations have been attributed to the vast differences in regional and political environments [12], cultural practices [13], health-seeking practices [14], and socioeconomic status [15] between these two areas.

For example, the majority of deceased children in the North were born to mothers who had no formal schooling and lived in rural areas, whereas many or most of the children who died in the South were born to mothers who had some secondary education and lived in urban areas. Adebowale et al. [16] previously suggested that this finding could be related to marrying early among young female adolescents in the northern region of Nigeria. Educated parents may be better equipped to offer their children appropriate care than parents with low levels of education. To rectify this situation, the Nigerian government has been pursuing policies aimed at improving access to education, especially among the poor in Nigeria.

Common wisdom suggests that health and illness or mortality generally follow the following social gradient: the lower the socioeconomic position, the worse the health. A higher proportion of households in the North than in the South belonged to the poorest wealth quintile, and the majority of the households in the South were in the two richest wealth quintiles. This finding is not unexpected and uncommon, because, as Adebowale et al. [16] put it, most women in the North, particularly those of childbearing age, do not actively participate in the labor force and are frequently less educated, while in the south, the high level of literacy may have eliminated most of the cultural attitudes against women receiving education and working outside the home [17].

Children born to mothers younger than 20 years of age were at greater risk of infant and under-five mortality. Factors contributing to this finding could include the mother’s physical immaturity, pregnancy complications, poor maternal nutritional status, inadequate use of maternal health services, and inexperience in child rearing [18, 19]

The onset of fatal illness occurred at a younger age among children in the South than in the North. In the South, the fatal illnesses were short in duration, and the majority of children died during the post-neonatal period (1–11 months of age), whereas in the North, the majority of children died between 24–59 months of age. These findings suggest that the presence of an epidemiologic transition in child mortality in the South, as the mortality rates observed in younger children increased as mortality levels in children aged under-five years declined [20]. However, we acknowledge that the hypothesized epidemiologic transition in the region warrants further investigation to fully document its impact on child survival in the region and in Nigeria as a whole. A companion paper on the Nigerian VASA study’s verbal autopsy findings has begun this exploration [21].

Based on the results of the computerized expert algorithms of the verbal autopsy (EAVA) analysis [21], malaria, pneumonia, and diarrhea were identified as the three leading causes of mortality among children aged 1–59 months in Nigeria; however, the ranking of the causes of death differed by age and region. In high malaria and pneumonia transmission and densely populated settings such as the southern region of Nigeria, deaths have been found to be concentrated in younger children with little immunity to malaria exists [21, 22]. In addition, the current study also revealed that exclusive breastfeeding decreased in the 6–11 month age group in the South. Previous studies have suggested that complementary foods are introduced and exclusive breast feeding decreases between the age of 6 and 11 months, changes that are accompanied by waning passive maternal immunity and loss of protective immunoglobulin from breast milk [23, 24]. These changes may, thereby, increase the risk of lower respiratory tract infections, including pneumonia [25, 26]. Understandably, because these young children are biologically frail, the course of illness is expected to proceed quickly, hence the shorter illness duration observed among deceased children in the South than among those in the North.

On the other hand, the deaths were more highly concentrated in the 24–59 month age group in the North, and a significantly higher proportion of deaths in that region were diarrhea-related [21]. Children in the 24–59 month age group have lost their passive immunity [22], and in the North, most of these children were born to poor families and, therefore, more likely to have a living situation with inadequate sanitation, unhygienic practices, many other children, and overcrowding, all of which might increase their risk of exposure to infectious agents [27, 28]. Additionally, people of low socioeconomic status may not be able to afford nutritious food, and inadequate nutrition has been recognized to suppress the immune system’s ability to fight off infections [29]. Data suggest that despite the Expanded Program on Immunization’s impact on child health, the burden of vaccine-preventable diseases, particularly in the most vulnerable infants, has remained high [30]. Access to healthcare, including vaccination, remained low particularly in the North.

The proportion of severely ill children was higher among those who did not seek care than those who sought care. This result was in accordance with the findings of an earlier study conducted in Bangladesh, as the perception of illness also played a role in decision-making in that study, the results of which suggested that those who were reportedly severely ill were thought to be less likely to survive and, therefore, did not seek care [31].

Many studies have also identified a negative association between delayed care seeking and child survival [32–34]. The results of our study revealed that although the vast majority of caregivers recognized signs or symptoms of possible/severe illness, formal care seeking was delayed among those who eventually sought care. Thus, the children were more ill by the time their caregivers decided to seek formal care, which, on average, was more than 24 hours after the onset of the illness. Active care seeking was delayed by another 24 hours, yielding an overall 2-day delay in seeking care from a formal provider, regardless of the perceived illness severity or region. The delay in deciding to seek care (or delay 1) often reflects a caregiver’s inability to recognize the gravity of the signs of childhood illness. Delays in deciding to seek care may also occur due to cultural traditions that encourage first seeking treatment at home and/or from a traditional healer [35]; additionally, this delay may suggest the presence of a disconnect between the mother’s recognition of illness signs and their perception of the importance/urgency of seeking healthcare [36]. Thus, perhaps it is no wonder that so many young children died while attempting to seek formal care or at the healthcare facility itself. Other authors have posited that past experience with similar illnesses and symptoms can motivate mothers to wait and see whether the illness subsides on its own, particularly in situations where the cost of care or transportation to seek healthcare are constraining factors, such as in Nigeria [37, 38].

The delay in formal care seeking did not vary significantly by the type of healthcare provider reached first. For example, the fact that caregivers delayed seeking care from community health workers just as much as they delayed seeking out other types of healthcare providers is striking, given Nigeria’s longstanding national effort to promote community health extension workers (CHEWs) as primary healthcare providers [39].

The current study also revealed that when some informal care was sought, approximately half (47.8%) of these informal providers consisted of pharmacists/drugs retailers, a finding that was more apparent in the South than in the North. These findings corroborate those of other studies. A census of the Nigerian patent and proprietary medicine vendors (PPMVs) carried out in 2013–14 [40] suggested that these shops were more accessible than health-care facilities, particularly in southern regions. However, the effectiveness of these drugs retailers in serving as a lay cadre in delivering basic health services has been found to vary, and further research may be warranted to identify and reinforce optimal PPMV training and knowledge. [41, 42]

The overall low rate of referral for moderately and severely sick children is also of concern. Of note, the referral rate was significantly higher among moderately to severely sick children in the South than among their counterparts in the North. Similarly low referral rates have been reported previously and attributed to healthcare workers desiring to maintain prestige among their patients, therefore being less likely to refer patients in need of higher-level care [43]. In the present study, 86.8% of the caregivers of referred children reported that the reason behind the referral was that the first health providers were not capable of managing their child’s illness, and approximately one-quarter mentioned that the facilities lacked basic supplies or equipment that could have improved their child’s condition. In other words, these children may have reached poorly functioning facilities with incompetent health staff and/or inappropriate or lacking treatments and equipment instead of accessing better services that, if accessed, may have prevented the child’s death [44]. The low referral rate may also have been due to both the caregivers and their sick children being failed by an overall dysfunctional health system [45]. In any case, the findings warrant additional study to understand the effect of dysfunctional health facilities on overall child mortality in the country. The reasons why children were referred or not referred is also worthy of further investigation from both the caregivers’ and the providers’ perspectives. Until such data are available, the country could benefit from the use of the tools and strategies developed by the World Health Organization (WHO) to monitor the quality of child healthcare in district hospitals on a continuous basis [45, 46]. Among the children who were moderately/severely sick upon discharge from the first health provider, those who went to another health provider lived longer than those who did not. In addition, the majority (82.5%) of moderately/severely sick children who were referred to a second health provider subsequently went. These positive findings reiterate the importance of the adoption and provision of referral practices from the primary to secondary and tertiary level health facilities for further management of sick children. The implementation of a well-functioning referral system and appropriate and timely referral of sick children will be essential if child mortality is to be reduced [47].

Existing interventions could prevent many deaths among ill children if they are utilized to promote appropriate and timely care [48]. Improving maternal recognition of certain signs and symptoms of childhood illness is critical and could change healthcare-seeking behavior [49, 50], thereby contributing to reduced child mortality in the country. To this end, the integrated management of childhood illness (IMCI) strategy aims to not only improve the providers’ skills in managing childhood illness but also to improve care-seeking behavior among families. In this strategy, healthcare workers are trained to teach mothers about danger signs and inform them about the need to seek care promptly if these signs occur [51]. Furthermore, one-half of avoidable child deaths in sub-Saharan Africa could be prevented through home-delivered interventions [52].

The social autopsy data also offered the opportunity to explore the following two sets of barriers that may offset the demand for healthcare: those that limit ability to consume and those that lower willingness to consume [53]. In the language of economics, these are constraints and preferences, respectively [53]. Inaccessibility to health facilities due to high costs, distance, and lack of transportation represents the most common constraint to formal healthcare seeking in both the northern and the southern regions of Nigeria [54, 55]. On the other hand, the most frequent barriers (preferences) in this study were ignorance of the severity of the child’s illness and use of traditional care in lieu of formal care. And these are factors that are influenced by culture, education, knowledge of the potential benefits of health care, and the quality of the services available [53].

Implementing and extending health insurance coverage has been included among the strategies that may be used to to promote access to health services, especially among the poor [52]. However, the challenges associated with this process are tremendous. For instance, according to O’Donnell [53], reforms need to be made to management, regulatory, and political mechanisms so that providers are motivated to deliver quality healthcare services.

One major limitation of this study was the absence of a control group, the inclusion of which would have allowed for testing whether significant differences existed in the coverage of interventions among cases (deceased children) and controls (live children). However, the lack of a comparison group in social autopsy studies is common and its inclusion may not be necessary since we are studying proven interventions that should be accessible to all pregnant mothers and newborns. Other limitations have been described elsewhere by Koffi et al. [9, 11].

## Conclusions

The results of previous studies and the findings of the current study illuminate the crucial role that maternal, household, and healthcare system factors play in child health and mortality. Cultural and educational factors may have hampered the recognition of illness and timely healthcare seeking, thereby limiting the potential benefits that may be derived from healthcare, while (economic) constraints may have suppressed utilization. The low rate of referral for moderately and severely sick children reflected a negative aspect of the health system. These findings suggest that interventions implemented to address child mortality in Nigeria can be divided into the following five basic categories: preventative measures such as anti-malaria drugs and immunization against specific pathogens; early recognition of and receipt treatment for illness; strengthening the referral system, especially in the North; educational attainment; and delayed marriage, the latter two of which may be especially important for women in the North. If the first three interventions fall within the purview of the healthcare system, the last two fall under the umbrella of a broader perspective of public health and require a multi-sectoral approach.

## Supporting information

S1 File Blank copy of the VASA Questionnaire given to study participants in Nigeria. (PDF)

S1 Fig Perceived illness severity score. (TIFF)
