# Supplementary material for: Beyond causes of death: The social determinants of mortality among children aged 1-59 months in Nigeria from 2009 to 2013
Source: PLoS One. 2017 May 31;12(5):e0177025. doi: 10.1371/journal.pone.0177025 (PMC5451019; doi:10.1371/journal.pone.0177025)
Supplement: S1 File — (PDF) [file pone.0177025.s001.pdf]

VERBAL/SOCIAL AUTOPSY GENERAL INFORMATION (FOR SBs, NN & CHILD DEATHS 0—59 MONTHS OLD)

Section 1: Background about the deceased

Interviewer: Before going to the field to do the interview, fill in this section from the survey or surveillance record for the deceased.

G1.1	Address of the household<br>*[Copy the household address]*	State ____________________________<br>LGA ______________________________<br>Locality ___________________________<br>EA _______________________________	☐☐☐<br>☐☐☐<br>☐☐☐☐<br>☐☐☐☐
	Directions to the household<br>*[Copy the directions to the household]*		
G1.2	Name of the deceased (if known)<br>*[Copy the name of the deceased]*		
G1.3	Sex of deceased<br>*[Copy the sex of the deceased]*	1. Male<br>2. Female	☐
G1.4	Date of birth of the deceased<br>*[Copy the day, month and year of birth of the deceased]*		___ ___/___ ___/___ ___ ___ ___<br>D D M M Y Y Y Y<br>*(DK = 99/99/9999)*
G1.5	Date of death of the deceased<br>*[Copy the day, month and year of death of the deceased]*		___ ___/___ ___/___ ___ ___ ___<br>*(DK = 99/99/9999)*
G1.6	Last known age of the deceased<br>*[Copy the last known age of the deceased: Record days if less than 28 days—if less than 24 hours, record "00" days; Record months if 28 days-11 months; Record years if 1 year or older.]*		___ ___ Days: ***1 or more → GQ1.7***<br>*(DK = 99)*<br>___ ___ Months → ***GQ1.7***<br>*(DK = 99)*<br>___ ___ Years → ***GQ1.7***<br>*(DK = 99)*
G1.6.1	Was this a stillbirth or neonatal death?<br>*[Copy this information from the record]*	1. Stillbirth<br>2. Neonatal death<br>9. Not known from the record	☐
G1.7	Name of mother<br>*[Copy the name of the mother]*		
G1.8	Name of father<br>*[Copy the name of the father]*		

Section 2: Background about the interview

Interviewer: Before and after the interview, fill in this section. These questions should not be asked of the respondent.

G2.1	Language of the interview		
G2.2	Interviewer name and ID number	________________________________	☐☐☐☐☐
G2.3	Dates of attempted and successful interviews	**DATE**	**RESULT OF THE INTERVIEW**
G2.3.1	Date of first interview attempt	__ __/__ __/__ __ __ __<br>D D M M Y Y Y Y	**Interim result:** ☐
G2.3.2	Date of second interview attempt	__ __/__ __/__ __ __ __<br>D D M M Y Y Y Y	**Interim result:** ☐
G2.3.3	Date of third interview attempt	__ __/__ __/__ __ __ __<br>D D M M Y Y Y Y	**Interim result:** ☐
G2.4	Date interview started<br>*[Equals date of the last attempt]*	__ __/__ __/__ __ __ __<br>D D M M Y Y Y Y	
G2.5	Time interview started<br>*[Record hour 1-24 / minutes 1-60]*	__ __/__ __<br>H R M M	
G2.6	Date interview finished<br>*[Equals date started or a later date]*	__ __/__ __/__ __ __ __<br>D D M M Y Y Y Y	**Final result:** ☐
G2.7	Time interview finished<br>*[Record hour 1-24 / minutes 1-60]*	__ __/__ __<br>H R M M	

Interview result codes:
1. Completed (Final result code)
2. Partially completed (Final result code)
3. Eligible respondent postponed interview
4. No eligible respondent at home at time of visit
5. Eligible respondent refused interview
6. No eligible respondent lives in household
7. No household member at home
8. Dwelling vacant / destroyed / not found
9. In progress (Interim result code)
10. Child reported dead in birth history is actually alive
11. Duplicate report of death – interview already conducted

G2.8	Date form checked by supervisor	__ __/__ __/__ __ __ __<br>D D M M Y Y Y Y	
G2.9	Date entered in computer	__ __/__ __/__ __ __ __<br>D D M M Y Y Y Y	

INTERVIEW BEGINS

Instructions to interviewer: Introduce yourself and explain the purpose of your visit. Ask to speak to the mother or to another adult who was the deceased's main caregiver during the illness that led to death. If this is not possible, arrange a time to revisit the household when the caregiver will be home. (See example below.)

"My name is [your name]. I am an interviewer with the ____________ project. I have been informed that a child death has occurred in your household. I am very sorry to hear this. Please accept my sympathies. For the purpose of improving health care, we are collecting information on recent child deaths in this area. I would like to talk to the mother or main caregiver of <NAME> and ask some questions about the events and any symptoms that <NAME> had during her/his illness before death."

Section 3: Consent

INTERVIEWER: Read the consent form to the respondent. Ask the respondent if he or she has any questions. Once any questions are answered, ask the respondent if he or she is willing to take part in the study.

G3.1	*INTERVIEWER: Did respondent give consent?*	1. Yes<br>2. No	☐ ***2 → Thank respondent for their time and end the interview.***

Section 4: Information about the respondent

Read: I would now like to ask you some general questions about yourself.

G4.1	What is your (the respondent's) name?		
G4.2	*INTERVIEWER: What is the sex of the respondent?*	1. Male<br>2. Female	☐
G4.3	What is your relationship to the deceased child?	1. Mother<br>2. Father<br>3. Grandmother<br>4. Grandfather<br>5. Aunt<br>6. Uncle<br>7. Brother<br>8. Sister<br>9. Birth attendant *(specify type)* ....<br>10. Other male *(specify)*.............................<br>11. Other female *(specify)*..........................	☐☐<br>__________<br>__________<br>__________
G4.4	How old are you?		___ ___ Years<br>*(DK = 99)*
G4.5	Starting with the first year of primary school, how many years of school did you complete?		___ ___ Years<br>*(<1 = 00; DK = 99)* ***>6 years → GQ4.6***
G4.5.1	Now I would like you to read this sentence to me. (*Show card to respondent)*<br><br>*If she cannot read the whole sentence, probe:* Can you read any part of the sentence to me?	1. Cannot read at all<br>2. Able to read only part of sentence<br>3. Able to read whole sentence<br>4. No card available to show mother	☐

Read: I would now like to ask you some questions about (your / the family's) household. Please remember that all information will be kept confidential.

[Read "...the family's household." if you are not conducting the interview at the household where the death was identified.]

G4.6	How many people live at (this / that) household?<br><br>*[Read "…at that address?" if you are speaking of "the family's household."]*		___ ___ People<br>*(DK = 99)*
G4.7	How many sleeping rooms are in the household?		___ ___ Rooms<br>*(DK = 99)*
G4.8	Does the household have a separate room for cooking?	1. Yes<br>2. No<br>9. Don't know	☐

G4.9	Does the household have:<br><br>*[Ask about each possession, and mark each one "Yes," "No" or "Don't know."]*	<u>Does the household have:</u><br>1. electricity?...........<br>2. a radio?...........<br>3. a television?...........<br>4. a refrigerator?...........<br>5. a fixed line telephone?...........<br><u>Does it have:</u><br>6. a mobile telephone?...........<br>7. a computer?...........<br>8. a bicycle?...........<br>9. a car or truck?...........<br><u>Does the household have:</u><br>10. piped water inside the residence?...........<br>11. piped water outside the residence?...........<br>12. a well (protected or unprotected)?...........<br>13. a water vendor, water supplied by truck or bottled water?...........<br>14. surface water?...........	<u>Yes</u> <u>No</u> <u>DK</u><br>1. ☐ 2. ☐ 9. ☐<br>1. ☐ 2. ☐ 9. ☐<br>1. ☐ 2. ☐ 9. ☐<br>1. ☐ 2. ☐ 9. ☐<br>1. ☐ 2. ☐ 9. ☐<br><br>1. ☐ 2. ☐ 9. ☐<br>1. ☐ 2. ☐ 9. ☐<br>1. ☐ 2. ☐ 9. ☐<br>1. ☐ 2. ☐ 9. ☐<br><br>1. ☐ 2. ☐ 9. ☐<br>1. ☐ 2. ☐ 9. ☐<br>1. ☐ 2. ☐ 9. ☐<br>1. ☐ 2. ☐ 9. ☐<br>1. ☐ 2. ☐ 9. ☐
G4.10	What type of toilet does the household have?	1. Flush toilet<br>2. Improved pit toilet<br>3. Traditional pit toilet<br>4. Bush/field/beach<br>5. Other *(specify)*...........<br>9. Don't know	☐<br><br>________________
G4.11	What is the main kind of energy the household uses for cooking?	1. Charcoal<br>2. Firewood<br>3. Kerosene<br>4. Electricity<br>5. Gas<br>6. Cow dung<br>7. Other *(specify)*...........<br>9. Don't know	☐<br><br>________________
G4.12	What is the main material used for the floor of the house?<br><br>*[If you are able to observe the floor, then mark the correct answer and do not ask this question.]*	1. Natural/mud<br>2. Cement<br>3. Wood<br>4. Tiles<br>5. Other *(specify)*...........<br>9. Don't know	☐<br><br>________________

Section 5: Information about others at the interview

G5.7	*INTERVIEWER: Are there other people present during the interview?*	1. Yes<br>2. No	☐ ***2 → GQ5.9***
G5.8	*INTERVIEWER: In addition to the respondent, how many people are present during the interview?*		___ ___ Other people<br>*(DK = 99)*
G5.9	*INTERVIEWER: Mark the respondent in the below table and whether s/he was present during the child's illness and/or death. For each other person present at the interview, ask the respondent their relationship to the deceased and whether they were present during the child's illness and/or at the death. For stillbirths and neonatal deaths, also ask if each person (other than the mother) was present during the mother's pregnancy and delivery.*		

	Relationship of person to the deceased child	Mark (X) if present at the interview	Stillbirths and neonatal deaths only: Present during the pregnancy: 1. Yes / 2. No	Stillbirths and neonatal deaths only: Present at the delivery: 1. Yes / 2. No	Neonatal & older child deaths only: Present during child's illness: 1. Yes / 2. No	Neonatal & older child deaths only: Present at the child's death: 1. Yes / 2. No
.1	Mother	☐			☐	☐
.2	Father	☐	☐	☐	☐	☐
.3	Grandmother	☐	☐	☐	☐	☐
.4	Grandfather	☐	☐	☐	☐	☐
.5	Aunt	☐	☐	☐	☐	☐
.6	Uncle	☐	☐	☐	☐	☐
.7	Brother	☐	☐	☐	☐	☐
.8	Sister	☐	☐	☐	☐	☐
.9	Traditional birth attendant	☐	☐	☐	☐	☐
.10	Other male (specify: ___________________)	☐	☐	☐	☐	☐
.11	Other female (specify ___________________)	☐	☐	☐	☐	☐

Study ID# | | | | | - | | | | - | | |
Village/Cluster HH Child

CHILD HEALTH EPIDEMIOLOGY REFERENCE GROUP
SB/NN/CHILD VERBAL/SOCIAL AUTOPSY QUESTIONNAIRE

**VA Section 1: Background (FOR STILLBIRTHS, NEONATAL & CHILD DEATHS 0—59 MONTHS OLD)**			
V1.1	Was the deceased a singleton or multiple birth?<br>*[If two or more children are born at the same time, it is counted as a multiple birth, even if one or more of the babies are born dead.]*	1. Singleton<br>2. Multiple<br>9. Don't know	☐ ***1 or 9 → VQ1.3***
V1.2	Was this the first, second, or later in the birth order?	1. First<br>2. Second<br>3. Third or more<br>9. Don't know	☐
V1.3	*If the mother is present, mark "Yes" and do not ask this question.*<br>Is the mother still alive?	1. Yes<br>2. No	☐ ***1 → VQ1.6***
V1.4	Did the mother die during or after the delivery?	1. During<br>2. After<br>9. Don't know	☐ ***1 or 9 → VQ1.6***
V1.5	How long after the delivery did the mother die?<br>*[Record days if less than 28 days—if less than 24 hours, record "00" days; Record months if 28 days or more]*		___ ___ Days<br>*(DK = 99)*<br>___ ___ Months<br>*(DK = 99)*
V1.6	Where was the deceased born?	1. Hospital<br>2. Other health provider or facility<br>3. On route to a health provider or facility<br>4. Home<br>5. Other *(specify)*.......................................<br>9. Don't know	☐<br>____________________________
V1.7	At the time of the delivery was the deceased:<br>*[Read the question and slowly read the first four choices. Respondent should hear all four choices & then respond.]*<br>*[Show photos]*	1. Very small<br>2. Smaller than usual<br>3. About average<br>4. Larger than usual<br>9. Don't know	☐
V1.8	What was the weight of the deceased at birth?		___ ___ ___ ___ Grams<br>*(DK = 9999)*
V1.9	What was the sex of the deceased?	1. Male<br>2. Female<br>9. Don't know	☐
V1.10	What was the delivery date?<br>*Compare the delivery date just stated by the respondent to the birth date from the prior record (GQ1.4). Discuss any inconsistency with the respondent to confirm or correct the stated delivery date. You cannot change the prior record's date.*		___ ___/___ ___/___ ___ ___ ___<br>D D M M Y Y Y Y<br>*(DK = 99/99/9999)*
V1.11	Was the child born alive or dead?	1. Alive<br>2. Dead<br>9. Don't know	☐
V1.12	Did the baby ever cry?	1. Yes<br>2. No<br>9. Don't know	☐

V1.13	Did the baby ever move?	1. Yes<br>2. No<br>9. Don't know	☐
V1.14	Did the baby ever breathe?	1. Yes<br>2. No<br>9. Don't know	☐
V1.15	*Refer to VQ1.11–1.14. If "Dead" & no crying, movement or breathing, mark "Stillbirth." If "Alive" & VQ1.12–1.14 = "No," or if "Dead" and VQ1.12, 1.13 or 1.14 = "Yes," then discuss & correct.*	1. Stillbirth<br>2. Live birth	☐ ***2 → VQ1.20***
**Stillbirths**			
V1.16	Were there any bruises or signs of injury on the baby's body at birth?	1. Yes<br>2. No<br>9. Don't know	☐
V1.17	Was the baby's body (skin and tissue) pulpy?	1. Yes<br>2. No<br>9. Don't know	☐
V1.18	Was any part of the baby physically abnormal at the time of delivery? (for example: body part too large or too small, additional growth on body)	1. Yes<br>2. No<br>9. Don't know	☐ ***2 or 9 → SQ3.1***
V1.19	What were the abnormalities?<br><br>*Ask for the following abnormalities [Mark all that apply – Show photos]*	1. Was the head size very small at the time of birth ....<br>2. Was the head size very large at the time of birth ....<br>3. Was there a mass defect on the back of head or spine....<br>4. Was there any other abnormality *(If "Yes," then specify)*....	Yes No<br>1. ☐ 2. ☐<br>1. ☐ 2. ☐<br>1. ☐ 2. ☐<br>1. ☐ 2. ☐ ___________
Inst_1: STOP. After completing VQ1.19 → SQ3.1 (Maternal history)			
**Live births**			
V1.20	How old was the child when the illness started?<br><br>*[Record days if less than 28 days—if less than 24 hours, record "00" days; Record months if 28 days-11 months; Record years if 1 year or older.]*		___ ___ Days *(DK = 99)*<br>___ ___ Months *(DK = 99)*<br>___ ___ Years *(DK = 99)*
V1.21	How long did the illness last?<br><br>*[Record days if less than 28 days—if less than 24 hours, record "00" days; Record months if 28 days or more.]*		___ ___ Days *(DK = 99)*<br>___ ___ Months *(DK = 99)*
V1.22	Where did the deceased die?	1. Hospital<br>2. Other health provider or facility<br>3. On route to a health provider or facility<br>4. Home<br>5. Other *(specify)*....<br>9. Don't know	☐<br>___________
V1.24	What was the date of death?<br><br>*Compare the date of death just stated by the respondent to the date of death from the prior record (GQ1.5). Discuss any inconsistency with the respondent to confirm or correct the stated date. You cannot change the prior record's date.*		___ ___/___ ___/___ ___ ___ ___<br>D D M M Y Y Y Y<br>*(DK = 99/99/9999)*

<table>
<tr><td rowspan="7">V1.25</td><td colspan="2">***AGE AT DEATH***

Record only the calculated age <u>OR</u> the stated age. First try to calculate the age. If this is not possible, then ask the respondent for the child's age at death.</td></tr>
<tr><td rowspan="3">CALCULATE THE AGE AT DEATH

Record the delivery date from VQ1.10: ___ ___/___ ___/___ ___ ___ ___
D D M M Y Y Y Y
(Don't Know = 99/99/9999)

Record the date of death from VQ1.24: ___ ___/___ ___/___ ___ ___ ___
D D M M Y Y Y Y
(Don't Know = 99/99/9999)

Now, if possible, calculate the age at death (VQ1.24 – VQ1.10). If only the month and year are known, you may still be able to calculate the approximate age in months or years. Discuss the calculated age with the respondent: I have calculated that the child was (about) <CALCULATED AGE> at death. Is this correct?

If the respondent does not agree with the calculated age, then again discuss the delivery date and date of death to make sure that these are correct. If the calculated age at death cannot be resolved, then go below to the “STATED AGE” box.

Once the age at death is calculated, check VQ1.20 and VQ1.21 to make sure that the age at illness onset and the illness duration are consistent with the age at death. For example, the age at onset + duration cannot be greater than the age at death.

[Record days if less than 28 days—if less than 24 hours, record “00” days; Record months if 28 days-11 months; Record years if 1 year or older.]

After recording the calculated age → VQ1.26</td><td>___ ___ Days (if < 28 days)
(DK = 99)</td></tr>
<tr><td>___ ___ Months (if 1-11 months)
(DK = 99)</td></tr>
<tr><td>___ ___ Years (if 1 year or older)
(DK = 99)</td></tr>
<tr><td rowspan="3">STATED AGE AT DEATH (Ask only if the calculated age cannot be determined)

How old was the deceased at the time of death?

Compare the age at death just stated by the respondent to the child's last known age from the prior record (GQ1.6). Discuss any inconsistency with the respondent to confirm or correct the stated age. You cannot change the prior record's age. Partly known delivery and death dates might help resolve the stated age. For example, if the child was born and died in the same month, then this is likely a neonatal death.

Once the age at death is determined, check VQ1.20 and VQ1.21 to make sure that the age at illness onset and the illness duration are consistent with the age at death. For example, the age at onset + duration cannot be greater than the age at death.

[Record days if less than 28 days—if less than 24 hours, record “00” days; Record months if 28 days-11 months; Record years if 1 year or older.]</td><td>___ ___ Days (if < 28 days)
(DK = 99)</td></tr>
<tr><td>___ ___ Months (if 1-11 months)
(DK = 99)</td></tr>
<tr><td>___ ___ Years (if 1 year or older)
(DK = 99)</td></tr>
<tr><td>V1.26</td><td>Mark the baby's age at the time of death.

[Use the calculated age (VQ1.24 – VQ1.10) if known, or the stated age (VQ1.25). If both the calculated and stated ages are unknown, then use your best judgment to mark the child's age at death.]

1. Less than 28 days old
2. 1-59 months old</td><td>☐ 2 → SQ5b.1</td></tr>
</table>

SA Module 3 and VA Section 2: Maternal history (FOR STILLBIRTHS AND NN DEATHS < 28 DAYS OLD)

Read: Now, I would like to ask you some questions about (your / the mother's) health and (your / her) pregnancy with <NAME>.

Here and in the following questions, read "…the mother…," "…her…" and "…she…" if the mother is not the respondent.

S3.1	Before the pregnancy with <NAME>, did (you / the mother) suffer from any of the following known conditions:<br><br>*[Read out all options and check "Yes," "No" or "Don't know" for each.]*<br><br>*If "Yes," then ask:* Did (you / she) undergo treatment for this condition during the pregnancy?	1. High blood pressure<br>2. Heart disease<br>3. Diabetes<br>4. Epilepsy/convulsion<br>5. Other<br><br>*(specify other)*............	Suffered from: Yes No DK / Treatment: Yes No DK<br>1. ☐ 2. ☐ 9. ☐ / 1. ☐ 2. ☐ 9. ☐<br>1. ☐ 2. ☐ 9. ☐ / 1. ☐ 2. ☐ 9. ☐<br>1. ☐ 2. ☐ 9. ☐ / 1. ☐ 2. ☐ 9. ☐<br>1. ☐ 2. ☐ 9. ☐ / 1. ☐ 2. ☐ 9. ☐<br>1. ☐ 2. ☐ 9. ☐ / 1. ☐ 2. ☐ 9. ☐<br><br>________________
S3.2	During the pregnancy, did (you / the mother) see anyone for antenatal care?	1. Yes<br>2. No<br>9. Don't know	☐ ***2 or 9 → SQ3.3***
S3.2.1	Whom did (you / she) see? Anyone else?<br><br>*[Probe, and record all persons seen.]*	1. Health care provider<br>2. TBA/Religious healer<br>3. Relative/neighbor/friend<br>4. Other *(specify)*<br>*(________________)*<br>9. Don't know	1. ☐<br>2. ☐<br>3. ☐<br>4. ☐<br>9. ☐<br>2, 3, 4, 9 → ***SQ3.3***
S3.2.2	How many times did (you / the mother) receive antenatal care from a health care provider during this pregnancy?		___ ___ Times<br>*(DK = 99)*
S3.2.3	During which month of the pregnancy did (you / the mother) last receive antenatal care from a health care provider?		___ ___ Month<br>*(DK = 99)*
S3.2.4	During this pregnancy, did the provider do any of the following for (you / the mother) at least once?<br><br>*[Read out all options and check "Yes," "No" or "Don't know" for each.]*<br><br>*[LOCAL ADAPTATION: Additional high energy and high protein foods to mention If the respondent asks]*	1. Did the provider measure (your / her) blood pressure?<br>2. Did (you / she) give a urine sample?<br>3. Did (you / she) give a blood sample?<br>4. Did the provider tell (you / her) to eat more high energy foods like <HIGH ENERGY FOODS> and high protein foods like <HIGH PROTEIN FOODS> than when not pregnant?<br>5. Did the provider tell (you / her) about the danger signs during pregnancy?<br>6. Did the provider tell (you / her) where to go if (you / she) had any danger signs?	Yes No DK<br>1. ☐ 2. ☐ 9. ☐<br>1. ☐ 2. ☐ 9. ☐<br>1. ☐ 2. ☐ 9. ☐<br>1. ☐ 2. ☐ 9. ☐<br>1. ☐ 2. ☐ 9. ☐<br>1. ☐ 2. ☐ 9. ☐
S3.3	Please tell me the danger signs during pregnancy or labor and delivery that you should seek care for immediately.<br><br>*Probe:* Tell me as many of the danger signs as you can.<br><br>*Probe:* Can you tell me any others?<br><br>*[Check each danger sign mentioned.]*	1. Vaginal bleeding<br>2. Convulsions/fits<br>3. Severe headache with blurred vision<br>4. Fever and too weak to get out of bed<br>5. Severe abdominal pain<br>6. Fast or difficult breathing<br>7. Painful contractions every 20 minutes or less for 12 hours or more<br>8. Broken water for 12 hours or more<br>9. Bloody, sticky discharge 12 hrs or more<br>10. No immediate danger sign mentioned	1. ☐<br>2. ☐<br>3. ☐<br>4. ☐<br>5. ☐<br>6. ☐<br>7. ☐<br>8. ☐<br>9. ☐<br>1–9 → ___ Mentioned<br>10. ☐
S3.4	During this pregnancy, (were you / was the mother) given an injection in the arm to prevent the baby from getting tetanus, that is, convulsions after birth?	1. Yes<br>2. No<br>9. Don't know	☐ ***2 or 9 → SQ3.5***

S3.4.1	During this pregnancy, how many times did (you / she) get this injection?		___ Times<br>*(DK = 9)*
S3.5	At any time before this pregnancy, did (you / the mother) receive any tetanus injection, either to protect yourself or another baby?	1. Yes<br>2. No<br>9. Don't know	☐ ***2 or 9 → SQ3.6***
S3.5.1	Before this pregnancy, how many other times did (you / she) receive a tetanus injection?<br><br>*[If 7 or more time, record "7."]*		___ Times<br>*(DK = 9)*
S3.6	*Skip SQ3.6-3.7.1 in areas wo/malaria.*<br><br>During this pregnancy, did (you / the mother) sleep under an insecticide treated bednet?	1. Yes, usually or always<br>2. Yes, sometimes<br>3. Never<br>9. Don't know	☐
S3.7	During this pregnancy, did (you / the mother) take any drug to prevent (you / her) from getting malaria?	1. Yes<br>2. No<br>9. Don't know	☐ ***2 or 9 → VQ2.1***
S3.7.1	During this pregnancy, how many times did (you / she) take this drug?		___ ___ Times<br>*(DK = 99)*
V2.1	Now I'd like to ask you about any problems (you / the mother) might have had during the pregnancy. Was the late part of the pregnancy (defined as the last 3 months), labor or delivery complicated by any of the following problems that started <u>before</u> the baby was delivered?<br><br>*[Read each complication and mark "Yes," "No" or "Don't know" for each.]*<br><br>*[Read "…the mother…" if the mother is not the respondent.]*	<u>Did (you / the mother) have</u>:<br>1. convulsions?<br>2. high blood pressure?<br>3. severe anemia or pallor <u>and</u> shortness of breath?<br>4. diabetes?<br>5. severe headache?<br>6. blurred vision?<br><u>(Were you / Was she)</u>:<br>7. too weak to get out of bed?<br><u>Did (you / the mother) have</u>:<br>8. severe abdominal pain?<br>9. fast or difficult breathing?<br>10. puffy face?<br>11. <u>any</u> vaginal bleeding before labor?<br>12. excessive bleeding during labor or delivery?<br>13. fever?<br>14. smelly vaginal discharge?<br><u>Was the:</u><br>15. child delivered not head first?<br>16. cord delivered first?<br>17. cord around the child's neck?<br><u>Did (you / the mother) have:</u><br>18. any other complication?<br><br>*(specify the other complication)*	<u>Yes</u> <u>No</u> <u>DK</u><br>1. ☐ 2. ☐ 9. ☐<br>1. ☐ 2. ☐ 9. ☐<br>1. ☐ 2. ☐ 9. ☐<br>1. ☐ 2. ☐ 9. ☐<br>1. ☐ 2. ☐ 9. ☐<br>1. ☐ 2. ☐ 9. ☐<br>1. ☐ 2. ☐ 9. ☐<br>1. ☐ 2. ☐ 9. ☐<br>1. ☐ 2. ☐ 9. ☐<br>1. ☐ 2. ☐ 9. ☐<br>1. ☐ 2. ☐ 9. ☐<br>1. ☐ 2. ☐ 9. ☐<br>1. ☐ 2. ☐ 9. ☐<br>1. ☐ 2. ☐ 9. ☐<br>1. ☐ 2. ☐ 9. ☐<br>1. ☐ 2. ☐ 9. ☐<br>1. ☐ 2. ☐ 9. ☐<br>1. ☐ 2. ☐ 9. ☐<br>___________________
V2.2A	Did (you / the mother) have any of the following problems that started <u>after</u> the delivery?<br><br>*[Read each complication and mark "Yes," "No" or "Don't know" for each.]*<br><br>*[Read "…the mother…" if the mother is not the respondent.]*	<u>Did (you / the mother) have</u>:<br>1. convulsions?<br>2. heavy bleeding?<br>3. Fever with smelly vaginal discharge or abdominal pain?	<u>Yes</u> <u>No</u> <u>DK</u><br>1. ☐ 2. ☐ 9. ☐<br>1. ☐ 2. ☐ 9. ☐<br>1. ☐ 2. ☐ 9. ☐
V2.2	How many months long was the pregnancy?		___ ___ Months ***≠ 99 → VQ2.4***<br>*(DK = 99)*

V2.3	Did the pregnancy end early, on time, or late?	1. Early<br>2. On time<br>3. Late<br>9. Don't know	☐
V2.4	Was the baby moving in the last few days before the birth?	1. Yes<br>2. No<br>9. Don't know	☐
V2.5	When did (you / the mother) last feel the baby move?<br>*[Read "…the mother…" if the mother is not the respondent.]*<br>*[Record hours if less than 24 hours; Record days if 1 day or more.]*		___ ___ Hours before delivery *(DK = 99)*<br>___ ___ Days before delivery *(DK = 99)*
V2.6	Did the water break before labor or during labor?<br>*[Note: Labor begins when contractions are no more than 20 minutes apart.]*	1. Before<br>2. During<br>9. Don't know	☐ ***2 or 9 → VQ2.8***
V2.7	How much time before labor did the water break?<br>*[Record "24" if 1 day or more.]*		___ ___ Hours *(DK = 99)*
V2.8	What was the color of the liquor when the water broke?	1. Green or brown<br>2. Clear (normal)<br>3. Other *(specify)*..<br>9. Don't know	☐<br>______________________________
V2.9	Was the liquor foul smelling?	1. Yes<br>2. No<br>9. Don't know	☐
V2.10	How much time did the labor and delivery take?<br>*[Record "00" if less than 1 hour.]*		___ ___ Hours *(DK = 99)*
S3.8	Where did the delivery occur?	1. Hospital<br>2. Other health provider or facility<br>3. On route to a health provider or facility<br>4. Home<br>*5.* Other *(specify____________________)*<br>9. Don't know	☐ 1-3 = Health provider<br>***9 → SQ3.11***
S3.9	Who decided that this was the right place to deliver the baby?<br>*[Record the one main decision maker.]*	1. The woman, herself<br>2. Her husband<br>3. Her mother<br>4. Her mother-in-law<br>5. Her father-in-law<br>6. Other *(specify)*......................................<br>9. Don't know	☐<br>_____________________________
S3.10	*If she did <u>not</u> go to a health provider or facility (SQ3.8 = 4-5) for the delivery, ask:* Did (you / the mother) have any concerns or problems that kept (you / her) from going to a health provider or facility for the delivery?<br>*If she <u>went</u> <u>or</u> <u>was</u> <u>on</u> <u>route</u> to a health provider or facility (SQ3.8 = 1-3) for the delivery, ask:* Did (you / the mother) have to overcome any concerns or problems to go to health provider or facility for the delivery?	1. Yes<br>2. No<br>9. Don't know	☐ ***2 or 9 → SQ3.11***

S3.10.1	What concerns or problems did (you / she) have?<br><br>*Prompt:* Was there anything else?<br><br>*[Multiple answers allowed.]*	1. Did not think she was sick enough to need health care....<br>2. No one available to go with her ....<br>3. Too much time from her regular duties...<br>4. Someone else had to decide *(specify)*....<br>5. Too far to travel ....<br>6. No transportation available....<br>7. Cost (transport, health care, other).........<br>8. Not satisfied with available health care...<br>9. Symptom(s) required traditional care......<br>10. Thought she was too sick to travel........<br>11. Thought she/baby will die despite care.<br>12. Was late at night (transportation or provider not available) ....<br>13. Fears exposure to male health provider<br>14. Other *(specify)*....<br>99. Don't know....	1. ☐<br>2. ☐<br>3. ☐<br>4. ☐ ____<br>5. ☐<br>6. ☐<br>7. ☐<br>8. ☐<br>9. ☐<br>10. ☐<br>11. ☐<br>12. ☐<br>13. ☐<br>14. ☐ ____<br>99. ☐
S3.11	Who (at the facility) delivered the baby?<br><br>*[Read "...at the facility..." if she delivered at a health facility.]*	1. Doctor<br>2. Nurse/midwife<br>3. Relative/neighbor/friend<br>4. Self (the mother)<br>5. Traditional birth attendant<br>6. Other *(specify)*....<br>9. Don't know	☐<br><br>____
S3.12	How soon after labor started did the <BIRTH ATTENDANT> first attend the mother?<br><br>*[Discuss that labor starts with painful contractions every 20 minutes or less.]*<br><br>*[Mark days &/or hours as needed: e.g. 00 day, 06 hours]*		___ ___ Days *(DK = 99)*<br><br>___ ___ Hours *(DK = 99)*
S3.13	Did the birth attendant use a pictorial graph to follow the progress of (your / the mother's) labor?	1. Yes<br>2. No<br>9. Don't know	☐
S3.14	Did the birth attendant wash her hands with soap and water or wear surgical gloves before assisting with the birth?	1. Yes, washed with soap and water<br>2. Yes, wore surgical gloves<br>3. No<br>9. Don't know	☐
S3.15	On what surface did (you / the mother) deliver?	1. Labor bed<br>2. Solid floor with mackintosh/cover<br>3. Solid washed floor<br>4. Solid unwashed floor<br>5. Dirt/soil/mud/straw floor<br>6. Other *(specify)*....<br>9. Don't know	☐<br><br>____
V2.17	Was the delivery...?<br><br>*[Read the choices and mark ONE.]*	1. Vaginal with forceps<br>2. Vaginal without forceps<br>3. Vaginal (don't know)<br>4. C-section<br>9. Don't know	☐
V2.18	During labor but before delivery, did (you / the mother) receive any kind of injection?<br><br>*[Read "…the mother…" if the mother is not the respondent.]*	1. Yes<br>2. No<br>9. Don't know	☐

SA Module 4: Careseeking for maternal complications (FOR STILLBIRTHS AND NN DEATHS < 28 DAYS OLD)

Read: Now, I would like to ask you some questions about (your / the mother's) careseeking during the pregnancy with <NAME>.

S4.1	**Maternal symptoms:**<br><br>*First look back at the maternal VA symptoms in VQ2.1 (options 1-14), 2.2, 2.7 and 2.10 (options 15-17). Mark ("X") these in the "Symptoms in the last 3 months" column.*<br><br>*If she had any symptom(s), then read:* Earlier, you mentioned that (you / the mother) had <SYMPTOM(S)> during the last 3 months of the pregnancy or during labor or delivery. Which of the symptoms started before labor? And which started with or during labor or delivery, including any that may have brought on the labor?<br><br>*[Remind the respondent that labor starts with painful contractions every 20 minutes or less. Then review each reported symptom with her to determine which started before labor and which started with or during labor or delivery. Do not include any symptoms here that started after the baby was delivered.]*		Symptoms during last 3 months<br>Yes	Started (related to labor/delivery)<br>Before W/D DK
		1. Convulsions	☐	1. ☐ 2. ☐ 9. ☐
		2. High blood pressure	☐	1. ☐ 2. ☐ 9. ☐
		3. Severe anemia or (pallor and SOB)	☐	1. ☐ 2. ☐ 9. ☐
		4. Diabetes	☐	1. ☐ 2. ☐ 9. ☐
		5. Severe headache	☐	1. ☐ 2. ☐ 9. ☐
		6. Blurred vision	☐	1. ☐ 2. ☐ 9. ☐
		7. Too weak to get out of bed	☐	1. ☐ 2. ☐ 9. ☐
		8. Severe abdominal pain (not labor pain)	☐	1. ☐ 2. ☐ 9. ☐
		9. Fast or difficult breathing	☐	1. ☐ 2. ☐ 9. ☐
		10. Puffy face	☐	1. ☐ 2. ☐ 9. ☐
		11. Any vaginal bleeding before labor	☐	1. ☐ 2. ☐ 9. ☐
		12. Excessive bleeding during labor or dlvr.	☐	1. ☐ 2. ☐ 9. ☐
		13. Fever	☐	1. ☐ 2. ☐ 9. ☐
		14. Smelly vaginal discharge	☐	1. ☐ 2. ☐ 9. ☐
		15. Early/preterm labor (less than 9 mnths)	☐	1. ☐ 2. ☐ 9. ☐
		16. Water broke 6 hrs or more before labor.	☐	1. ☐ 2. ☐ 9. ☐
		17. Labor for 12 hours or more	☐	1. ☐ 2. ☐ 9. ☐
		18. Other *(specify)*<br>(______________________)	☐	1. ☐ 2. ☐ 9. ☐
		19. No symptoms during last 3 months	☐ → ***Inst_8***	
		20. No symptoms before labor		☐ → ***SQ4.11***
S4.2	Did (you / the mother) seek care from any person or health facility for (any of) the pregnancy symptom(s) that started before labor?<br><br>*[Read "…for any of…" if she had more than one pregnancy symptom.]*	1. Yes<br>2. No<br>9. Don't know	☐ ***2 → SQ4.4***<br>***9 → Inst_2***	
S4.2.1	Where did (you / she) seek this care?<br><br>*Prompt:* Was there anywhere else?<br><br>*[Multiple answers allowed.]*	1. Hospital<br>2. NGO or government clinic<br>3. Private doctor/clinic<br>4. Community nurse or midwife<br>5. TBA/village doctor/quack/other non-formal or traditional provider<br>6. Relative, neighbor or friend<br>7. Other *(specify)*<br>(______________________)<br>99. Don't know	1. ☐ 2. ☐ 3. ☐ 4. ☐ → = Health provider<br>5. ☐ 6. ☐ 7. ☐ → ***SQ4.4***<br>9. ☐ → ***Inst_2***	
S4.3	*If more than one symptom started before labor and she sought care from a health provider (SQ4.2.1 = 1-4), ask:* For which symptom or symptoms that started before labor did (you / she) seek care from a health provider or facility?	1. Convulsions ☐<br>2. High blood pressure ☐<br>3. Severe anemia or (pallor and SOB) ☐<br>4. Diabetes ☐<br>5. Severe headache ☐<br>6. Blurred vision ☐<br>7. Too weak to get out of bed ☐<br>8. Severe abdominal (not labor) pain ☐<br>9. Fast or difficult breathing ☐	10. Puffy face ☐<br>11. Any bleeding before labor ☐<br>12. – blank –<br>13. Fever ☐<br>14. Smelly vaginal discharge ☐<br>15. – blank –<br>16. Water broke ≥6 hrs bfr. labor ☐<br>17. – blank –<br>18. Other *(specified in SQ4.1)* ☐	

S4.4	*If she never went to a health provider (SQ4.2 = 2 or SQ4.2.1 ≠ 1-4) for any of the pregnancy symptoms, ask:* Did (you / the mother) have any concerns or problems that kept (you / her) from going to a health provider or facility for the symptom(s) that started before labor?<br><br>*If she went to health provider (SQ4.2.1 = 1-4) for any pregnancy symptom(s), ask:* Did (you / the mother) have to overcome any concerns or problems to go to a health provider or facility for the symptom(s) that started before labor?	1. Yes<br>2. No<br>9. Don't know	☐ ***2 or 9 → Inst_1***
S4.4.1	What concerns or problems did (you / she) have?<br><br>*Prompt:* Was there anything else?<br><br>*[Multiple answers allowed.]*	1. Did not think was sick enough to need health care........<br>2. No one available to go with her ....<br>3. Too much time from her regular duties...<br>4. Someone else *(specify)* had to decide....<br>5. Too far to travel ....<br>6. No transportation available........<br>7. Cost (transport, health care, other)........<br>8. Not satisfied with available health care...<br>9. Symptom(s) required traditional care......<br>10. Thought she was too sick to travel........<br>11. Thought she/baby will die despite care.<br>12. Fears exposure to male health provider<br>13. Other *(specify)*........<br>99. Don't know........	1. ☐<br>2. ☐<br>3. ☐<br>4. ☐ ______<br>5. ☐<br>6. ☐<br>7. ☐<br>8. ☐<br>9. ☐<br>10. ☐<br>11. ☐<br>12. ☐<br>13. ☐ ______<br>99. ☐
Inst_1: If SQ4.2 = 2 or SQ4.2.1 ≠ 1-4 (Never went to a health provider for any pregnancy symptoms) → Inst_2			
S4.5	Did any health provider or facility refer (you / her) to another health provider or facility for (any of) the symptom(s) that started before labor?	1. Yes<br>2. No<br>9. Don't know	☐ ***2 or 9 → SQ4.6***
S4.5.1	Did (you / she) go to the provider or facility to which (you were / she was) referred?	1. Yes<br>2. No<br>9. Don't know	☐
S4.6	How many different health providers or facilities did (you / the mother) see for the pregnancy symptom(s) that started before labor?		___ ___ Health providers/facilities<br>*(DK = 99)*
S4.7	(Were you / Was the mother) admitted to hospital for (any of) the symptom(s) that started before labor?	1. Yes<br>2. No<br>9. Don't know	☐
S4.8	Please tell me everything that the provider(s) suggested that (you / the mother) do for the pregnancy symptom(s) at home?<br><br>*Prompt:* Was there anything else?<br><br>*[Multiple answers allowed.]*	1. Take antibiotic by mouth........<br>2. Take antimalarial by mouth ....<br>3. Take BP medicine by mouth........<br>4. Take other medicine by mouth ....<br>5. Rest / bed rest / decrease work........<br>6. Return for follow-up visit(s)........<br>7. Return or referred if worse........<br>8. Other *(specify)*........<br>9. Nothing........<br>99. Don't know........	1. ☐<br>2. ☐<br>3. ☐<br>4. ☐<br>5. ☐<br>6. ☐<br>7. ☐<br>8. ☐ ______<br>9. ☐ ***→ Inst_2***<br>99. ☐ ***→ Inst_2***
S4.9	(Were you / Was the mother) able to follow all this advice?	1. Yes<br>2. No<br>9. Don't know	☐ ***9 → Inst_2***

S4.10	*If not able to follow all the advice, ask:* Did (you / she) have any concerns or problems that kept (you / her) from following the advice?<br><br>*If able to follow all the advice, ask:* Did (you / she) have to overcome any concerns or problems to follow the advice?	1. Yes<br>2. No<br>9. Don't know	☐ ***2 or 9 → Inst_2***
S4.10.1	What concerns or problems did (you / she) have?<br><br>*Prompt:* Was there anything else?<br><br>*[Multiple answers allowed.]*	1. Did not understand instructions.............<br>2. Too much time from her regular duties...<br>3. Someone else *(specify)* decided ....<br>4. Cost too much ....<br>5. Problem required traditional care ....<br>6. Advised care not needed or helpful........<br>7. Advised care might harm unborn child ...<br>8. Thought she/baby will die despite care ..<br>9. Other *(specify)*.......................................<br>99.Don't know..	1. ☐<br>2. ☐<br>3. ☐ ______________________<br>4. ☐<br>5. ☐<br>6. ☐<br>7. ☐<br>8. ☐<br>9. ☐ ______________________<br>99. ☐
Inst_2: Refer to SQ4.1: If no labor or delivery symptoms → Inst_8			
S4.11	Now let's talk about the labor and delivery symptom(s). You said earlier that the symptom(s) that started with or during labor or delivery (was / were) <SYMPTOM(S)>.<br><br>*[Read and mark the SQ4.1 symptom(s) confirmed by the respondent. Correct the SQ4.1 responses if necessary.]*	1. Convulsions...................................... ☐<br>2. High blood pressure .... ☐<br>3. Severe anemia or (pallor and SOB) .. ☐<br>4. – blank –<br>5. Severe headache .... ☐<br>6. Blurred vision.................................... ☐<br>7. Too weak to get out of bed .... ☐<br>8. Severe abdominal (not labor) pain .... ☐<br>9. Fast or difficult breathing .... ☐	10.Puffy face .... ☐<br>11.Any bleeding before labor .... ☐<br>12.Excess bleed during L or D ..... ☐<br>13.Fever...................................... ☐<br>14.Smelly vaginal discharge .... ☐<br>15.Early/preterm labor (<9 mnth) . ☐<br>16.Water broke ≥6 hrs bfr. labor .. ☐<br>17.Labor for 12 hours or more ..... ☐<br>18.Other *(specified in SQ4.1)*....... ☐
S4.12	Where (were you / was the mother) when (this / the first) symptom began?<br><br>*[Read "…the first…" if she had more than one labor or delivery symptom.]*	1. Home<br>2. On route to a health provider or facility<br>3. At the health provider or facility where she went for normal labor<br>4. Other *(specify)*.......................................<br>9. Don't know	☐ ***3 → SQ4.17***<br><br>___________________________
S4.13	Did (you / she) ever receive, seek or try to seek any care or treatment for (any of) the labor or delivery symptom(s)?<br><br>*[Read "…any of the symptoms" if she had more than one symptom.]*	1. Yes<br>2. No<br>9. Don't know	☐ ***2 → SQ4.14***<br>***9 → SQ4.17***
S4.13.1	What was the first thing (you / she) did for the symptom(s)?<br><br>*[Mark only the first action taken.]*	1. Home treatment (at her own home, or by a relative, neighbor, or friend)<br>Sought or tried to seek care from a:<br>2. Hospital<br>3. NGO or government clinic<br>4. Private doctor/clinic<br>5. Community nurse or midwife<br>6. Pharmacist or drug seller<br>7. TBA/village doctor/quack/other non-formal or traditional provider<br>8. Other *(specify)* ....<br>99. Don't know	☐☐ ***99 → SQ4.16***<br><br>__________________________
S4.14	Who decided that this was the right thing to do (at that time)?<br><br>*[Read "…at that time" if she received or sought any care or treatment.]*<br><br>*[Only one response allowed. Record the main decision maker.]*	1. The woman, herself<br>2. Her husband<br>3. Her mother<br>4. Her mother-in-law<br>5. Her father-in-law<br>6. Other *(specify)*......................................<br>9. Don't know	☐<br><br>___________________________

S4.15	*If she did not go to a health provider (SQ4.13 = 2 or SQ4.13.1 = 1 or 6-8), ask:* Did (you / the mother) have any concerns or problems that kept (you / her) from going to a health provider (at that time)?<br><br>*Do not read "…at that time?" if she never received or sought any care or treatment (SQ4.13 = 2).*<br><br>*If she went to a health provider (SQ4.13.1 = 2-5), ask:* Did (you / the mother) have to overcome any concerns or problems to go to the <HEALTH PROVIDER> at that time?	1. Yes<br>2. No<br>9. Don't know	☐ ***2 or 9 →***<br>***Inst_2.5 (if SQ4.13 = 2), or Inst_3 (if SQ4.13.1 = 2-5)***
S4.15.1	What concerns or problems did (you / she) have?<br><br>*Prompt:* Was there anything else?<br><br>*[Multiple answers allowed.]*	1. Did not think she was sick enough to need health care....<br>2. No one available to go with her ....<br>3. Too much time from her regular duties...<br>4. Someone else had to decide *(specify)*....<br>5. Too far to travel ....<br>6. No transportation available....<br>7. Cost (transport, health care, other)....<br>8. Not satisfied with available health care...<br>9. Symptom(s) required traditional care......<br>10. Thought she was too sick to travel.......<br>11. Thought she/baby will die despite care.<br>12. Was late at night (transportation or provider not available) ....<br>13. Fears exposure to male health provider<br>14. Other *(specify)* ....<br>99. Don't know....	1. ☐<br>2. ☐<br>3. ☐<br>4. ☐ ____________<br>5. ☐<br>6. ☐<br>7. ☐<br>8. ☐<br>9. ☐<br>10. ☐<br>11. ☐<br>12. ☐<br>13. ☐<br>14. ☐ ____________<br>99. ☐
Inst_2.5: If SQ4.13 = 2 → SQ4.17<br>***Inst_3: If SQ4.13.1 = 2-5 (First went to a health provider or facility) → SQ4.16.1***			
S4.16	Did (you / she) ever seek or try to seek care from a health provider or facility for (any of) the labor or delivery symptom(s)?	1. Yes<br>2. No<br>9. Don't know	☐ ***2 or 9 → SQ4.17***
S4.16.1	Please tell me all the types of health providers and facilities where (you / she) sought or tried to seek care for (any of) the labor or delivery symptom(s).<br><br>*Prompt:* Anywhere else?<br><br>*[Multiple answers allowed.]*	1. Hospital ....<br>2. NGO or government clinic ....<br>3. Private doctor/clinic ....<br>4. Community nurse or midwife ....<br>9. Don't know....	1. ☐<br>2. ☐<br>3. ☐<br>4. ☐<br>9. ☐
S4.17	*Refer to SQ3.8 to determine the delivery place. Discuss with respondent to confirm or correct the delivery place.*<br><br>*Discuss & resolve inconsistencies, for example, if SQ4.13 or 4.16 = "No," but the mother delivered in a health facility.*	1. Hospital<br>2. Other health provider or facility<br>3. On route to a health provider or facility<br>4. Home<br>5. Other *(specify)*....<br>9. Don't know	☐ 1-3 = Health provider<br><br>____________
S4.18	So, including where (you / the mother) went or tried to go for the labor or delivery symptom(s) and for the delivery, how many health providers or facilities did (you / she) go to?<br><br>*[If SQ4.16 = 2 and SQ4.17 = 4 or 5 → record '00' health providers/facilities]*<br>*[If SQ4.16 = 2 and SQ4.17 = 1-3 → record '01' health provider/facility]*<br>*[If SQ4.16 = 2 and SQ4.17 = 9 → record '99' health providers/facilities]*<br>*[If SQ4.16 = 9 → record '99' health providers/facilities]*		___ ___ Health providers/facilities

Inst_4: If SQ4.12 = 3 (Symptoms began at the health provider where she went for normal labor) → SQ4.22

Inst_5: If SQ4.16 = 2 or 9 & SQ4.17 = 4-9 (No health provider seen/sought for the symptoms/delivery) → Inst_8

Inst_5.5: If SQ4.1 = only 1 labor or delivery symptom OR If SQ4.16 = 2 or 9 → SQ4.21

S4.19	Was there any particular symptom or symptoms for which (you / the mother) went to the (first) health provider?<br><br>*[Read "…the first health provider?" if she went to more than one provider.]*	1. Yes<br>2. No<br>9. Don't know	☐ *2 or 9 → SQ4.21*
S4.20	For which symptom(s) did (you / she) go?	1. Convulsions ☐<br>2. High blood pressure ☐<br>3. Severe anemia or (pallor and SOB) ☐<br>4. – blank –<br>5. Severe headache ☐<br>6. Blurred vision ☐<br>7. Too weak to get out of bed ☐<br>8. Severe abdominal (not labor) pain ☐<br>9. Fast or difficult breathing ☐	10. Puffy face ☐<br>11. Any bleeding before labor ☐<br>12. Excess bleed during L or D ☐<br>13. Fever ☐<br>14. Smelly vaginal discharge ☐<br>15. Early/preterm labor (<9 mnth) ☐<br>16. Water broke ≥6 hrs bfr. labor ☐<br>17. Labor for 12 hours or more ☐<br>18. Other *(specified in SQ4.1)* ☐
S4.21	How long after the labor or delivery symptom(s) began was it decided to go to the (first) health provider?<br><br>*[Read "…to the first…" if she went or tried to go to more than one health provider.*<br><br>*[Mark days, hours &/or minutes as needed: e.g. 00 day, 02 hours, 10 minutes]*		___ ___ Days *(DK = 99)*<br><br>___ ___ Hours *(DK = 99)*<br><br>___ ___ Minutes *(DK = 99)*

Labor and delivery matrix instructions: *Ask the following questions for the first and last health providers where she sought/tried to seek care for the labor and delivery symptoms. If she delivered at a health provider/facility or at home or on route while trying to go to a health provider/facility, then that should be the first health provider (if she went to only one) or the last health provider. Ask all the questions for the first provider before going on to the last.*

Before asking about the first health provider, read:
Now I would like to ask about (your / the mother's) visit to the (first) health provider. *[Read "first" if she went or tried to go to more than one provider.]*

Before asking about the last health provider, read:
Now I would like to ask about (your / the mother's) visit to the last health provider.

**– LABOR AND DELIVERY MATRIX QUESTIONS –**		**FIRST HEALTH PROVIDER**	**LAST HEALTH PROVIDER**
What was the name of the (first / last) health provider or facility where (you / the mother) (sought care for the labor or delivery symptom(s) / delivered the baby / tried to deliver the baby)?<br><br>*Probe to identify the type of provider.*	1. Hospital (Government)<br>2. Hospital (NGO)<br>3. Hospital (Private)<br>4. Health center (Government)<br>5. Health center (NGO)<br>6. Health post (Government)<br>7. Health post (NGO)<br>8. Private doctor/clinic (Formal)<br>9. Private doctor/clinic (?Formal?)<br>10. Trained community nurse/midwife<br>99. Don't know	S4.22<br>☐☐<br><br>__________<br>(Name of Provider/Facility)	S4.32<br>☐☐<br><br>__________<br>(Name of Provider/Facility)
After (deciding to seek care / being referred), how much time passed before going to the <FIRST/LAST HEALTH PROVIDER>?<br><br>*[Discuss that this might include the time needed to arrange for transportation and money to go to the provider/facility, or to provide home care or go to a traditional provider before going to the health provider.]*		S4.23<br>___ ___ Days *(DK = 99)*<br><br>___ ___ Hours *(DK = 99)*	S4.33<br>___ ___ Days *(DK = 99)*<br><br>___ ___ Hours *(DK = 99)*

[If she delivered at home, record the time from decision/referral to delivery.]<br><br>*[Mark days, hours &/or minutes as needed: e.g. 00 days, 02 hours, 10 minutes]*		___ ___ Minutes<br>*(DK = 99)*	___ ___ Minutes<br>*(DK = 99)*
Was there any cost to travel to the <FIRST/LAST HEALTH PROVIDER> or pay for (your / the mother's) care there?	1. Yes<br>2. No<br>9. Don't know	S4.24<br>☐ ***2 or 9 → SQ4.25***	S4.34<br>☐ ***2 or 9 → SQ4.35***
How did (you / the mother) arrange for the money for these expenses?<br><br>*[Multiple answers allowed.]*	1. Had available ....<br>2. Borrowed......................................<br>3. Sold assets ....<br>4. Help from kin/relatives..................<br>5. Community fund...........................<br>6. Govt. scheme...............................<br>7. Other..<br>9. Don't know	S4.24.1<br>1. ☐<br>2. ☐<br>3. ☐<br>4. ☐<br>5. ☐<br>6. ☐<br>7. ☐<br>9. ☐	S4.34.1<br>1. ☐<br>2. ☐<br>3. ☐<br>4. ☐<br>5. ☐<br>6. ☐<br>7. ☐<br>9. ☐
What transportation method was used to go there?<br><br>*[Multiple answers allowed.]*	1. Walk...<br>2. Bicycle/animal/cart/boat ....<br>3. Bus...<br>4. Taxi/auto/trecker/motorcycle ....<br>5. Ambulance ....<br>6. Other...<br>7. Could not arrange transport ....<br>9. Don't know	S4.25<br>1. ☐ ***If only walk***<br>2. ☐ ***→ SQ4.26.1***<br>3. ☐<br>4. ☐<br>5. ☐<br>6. ☐<br>7. ☐ ***→ SQ4.26.1***<br>9. ☐	S4.35<br>1. ☐ ***If only walk***<br>2. ☐ ***→ SQ4.36.1***<br>3. ☐<br>4. ☐<br>5. ☐<br>6. ☐<br>7. ☐ ***→ SQ4.36.1***<br>9. ☐
How much did the transportation cost?		S4.26<br>____ ____ ____ ____ unit<br>*(DK = 9999)*	S4.36<br>____ ____ ____ ____ unit<br>*(DK = 9999)*
Did (you / the mother) reach the <FIRST/LAST HEALTH PROVIDER> before delivering the baby?<br><br>*If "No," discuss with respondent to reach correct response: 2, 3 or 4.]*	1. Yes, reached before delivering<br>2. No, delivered before setting out<br>3. No, delivered on route to provider<br>4. No, could not reach this provider – did not set out/returned home/took other action<br>9. Don't know	S4.26.1<br>☐ ***2, 3 → Inst_8***<br>***4, 9 → Inst_7***	S4.36.1<br>☐ ***2-9 → Inst_8***
How long did it take to travel to the <FIRST/LAST HEALTH PROVIDER>?<br><br>*[Mark hours &/or minutes as needed: e.g. 05 hours, 30 minutes]*		S4.27<br>____ ____ Hours<br>*(DK = 99)*<br><br>____ ____ Minutes<br>*(DK = 99)*	S4.37<br>____ ____ Hours<br>*(DK = 99)*<br><br>____ ____ Minutes<br>*(DK = 99)*

What did the <FIRST/LAST HEALTH PROVIDER> do for (your / the mother's) (labor or delivery symptom(s) / delivery)?<br><br>*Prompt:* Was there anything else?<br><br>*[Multiple answers allowed.]*	1. Gave oxygen for the baby<br>2. Gave antibiotics by mouth<br>3. Gave antimalarial by mouth<br>4. Gave BP medicine by mouth<br>5. Other medicine by mouth *(specify)*<br>6. Gave medicine to stop bleeding<br>7. Gave medicine to stop convulsions<br>8. Gave medicine to strengthen labor<br>9. Gave medicine to stop labor<br>10. Gave medicine for baby's lungs<br>11. Gave IM medicine<br>12. Gave IV fluids or medicine<br>13. Blood transfusion<br>14. Advised to buy outside medicine<br>15. Uterine massage<br>16. Did a C-section<br>17. Did another operation *(specify)*<br><br>18. Admitted to hospital<br><br>19. Other *(specify)*<br>20. Nothing<br>99. Don't know	S4.28<br>1. ☐<br>2. ☐<br>3. ☐<br>4. ☐<br>5. ☐ ____________<br>6. ☐<br>7. ☐<br>8. ☐<br>9. ☐<br>10. ☐<br>11. ☐<br>12. ☐<br>13. ☐<br>14. ☐<br>15. ☐<br>16. ☐<br>17. ☐ ____________<br><br>18. ☐ stayed __ __ days<br><br>19. ☐ ____________<br>20. ☐ → ***SQ4.30***<br>99. ☐ → ***SQ4.30***	S4.38<br>1. ☐<br>2. ☐<br>3. ☐<br>4. ☐<br>5. ☐____________<br>6. ☐<br>7. ☐<br>8. ☐<br>9. ☐<br>10. ☐<br>11. ☐<br>12. ☐<br>13. ☐<br>14. ☐<br>15. ☐<br>16. ☐<br>17. ☐ ____________<br><br>18. ☐ stayed __ __ days<br><br>19. ☐ ____________<br>20. ☐ → ***SQ4.40***<br>99. ☐ → ***SQ4.40***
How much did (you / the mother) pay for these treatments and other costs related to the health care, including any admission fee, consultation, lab tests, equipment, and room and food for companions?		S4.29<br>___ ___ ___ ___ ___ unit<br>*(DK = 99999)*	S4.39<br>___ ___ ___ ___ ___ unit<br>*(DK = 99999)*
Did the <FIRST/LAST HEALTH PROVIDER> refer (you / the mother) to another health provider or facility?	1. Yes<br>2. No<br>9. Don't know	S4.30<br>☐ ***2 or 9 → SQ4.30.2***	4.40<br>☐ ***2 or 9 → SQ4.40.2***
Why (were you / was the mother) referred?<br><br>*[Multiple answers allowed.]*	1. The provider was not capable of managing the problem<br>2. Required supplies (e.g., drugs, IV, oxygen, blood) not available<br>3. Required equipment (e.g., ultrasound) not available<br>4. Required facility (e.g., operation room) not available<br>9. Don't know	S4.30.1<br>1. ☐<br>2. ☐<br>3. ☐<br>4. ☐<br>9. ☐	S4.40.1<br>1. ☐<br>2. ☐<br>3. ☐<br>4. ☐<br>9. ☐
Was the baby delivered at the <FIRST/LAST HEALTH PROVIDER>?	1. Yes<br>2. No<br>9. Don't know	S4.30.2<br>☐ ***1 → Inst_8***	S4.40.2<br>☐ ***1 → Inst_8***
Inst_6: Check SQ4.18 to determine if she went to another health provider			
If did not go to another health provider, ask: Did (you / the mother) have any concerns or problems that kept (you / her) from going to another provider?<br><br>*If went to another health provider, ask:* Did (you / the mother) have to overcome any concerns or problems to go to another provider?	1. Yes<br>2. No<br>9. Don't know	S4.31<br>☐ ***2 or 9 → Inst_7***	S4.41<br>☐ ***2 or 9 → Inst_8***

What concerns or problems did (you / she) have?<br><br>*Prompt:* Was there anything else?<br><br>*[Multiple answers allowed.]*	1. Thought no more care needed.......<br>2. No one available to go with her......<br>3. Too much time from regular duties.<br>4. Someone else *(specify)* decided ....<br>5. Too far to travel ....<br>6. No transportation available............<br>7. Cost (transport, health care, other)<br>8. Not satisfied with available care .....<br>9. Problem required traditional care ...<br>10. Thought too sick to travel............<br>11. Thought she/baby will die anyway<br>12. Was late at night ....<br>13. She delivered before going..........<br><br>14. Other *(specify)* ....<br>99. Don't know	S4.31.1<br>1. ☐<br>2. ☐<br>3. ☐<br>4. ☐ ______________<br>5. ☐<br>6. ☐<br>7. ☐<br>8. ☐<br>9. ☐<br>10. ☐<br>11. ☐<br>12. ☐<br>13. ☐ → ***Inst_8***<br><br>14. ☐ ______________<br>99. ☐	S4.41.1<br>1. ☐<br>2. ☐<br>3. ☐<br>4. ☐ ______________<br>5. ☐<br>6. ☐<br>7. ☐<br>8. ☐<br>9. ☐<br>10. ☐<br>11. ☐<br>12. ☐<br>13. ☐<br><br>14. ☐ ______________<br>99. ☐
Inst_7: Check SQ4.18 → If she went to another health provider		***…go to SQ4.32 (LAST HEALTH PROVIDER)***	
Inst_8: STOP – If VQ1.15 = 1 (Stillbirth) → VQ5.4 (Section 5: Health records)			

SA Module 5a: Care of the newborn; and VA Section 3: Neonatal deaths (FOR NN DEATHS <28 DAYS OLD)

Read: Now I would like to ask you about the care of the newborn child.

S5a.1	What tool was used for cutting the cord?	1. New/from delivery kit/boiled razor blade<br>2. Old razor blade<br>3. Scissors<br>4. Other *(specify)*......................................<br>9. Don't know	☐<br>________________
S5a.2	What material was used for tying the cord?	1. Clean/from delivery kit/boiled piece of thread<br>2. Unclean piece of thread<br>3. Cord clamp<br>4. Other *(specify)*......................................<br>9. Don't know	☐<br>________________
S5a.3	Was anything applied to the umbilical cord stump after birth?	1. Yes<br>2. No<br>9. Don't know	☐ ***2 or 9 → VQ3.1***
S5a.3.1	What was it?	1. Alcohol/other antiseptic<br>2. Antibiotic ointment/cream/powder<br>3. Castor oil, mustard oil or shea butter<br>4. Animal dung or dirt/mud<br>5. Other *(specify)*.....................................<br>9. Don't know	☐<br>________________
V3.1	Were there any bruises or signs of injury on the baby's body at birth?	1. Yes<br>2. No<br>9. Don't know	☐
V3.2	Was any part of the baby physically abnormal at the time of delivery? (for example: body part too large or too small, additional growth on body)	1. Yes<br>2. No<br>9. Don't know	☐ ***2 or 9 → VQ3.4***
V3.3	What were the abnormalities?<br><br>*Ask for the following abnormalities: [Mark all that apply – Show photos]*	1. Was the head size very small at the time of birth? ....<br>2. Was the head size very large at the time of birth? ....<br>3. Was there a mass defect on the back of head or spine?......................................<br>4. Was there any other abnormality?..........<br><br>*(If "Yes," then specify)*	Yes No<br>1. ☐ 2. ☐<br>1. ☐ 2. ☐<br>1. ☐ 2. ☐<br>1. ☐ 2. ☐<br>________________
V3.4	Did the baby breathe immediately after birth?	1. Yes<br>2. No<br>9. Don't know	☐ ***2 → VQ3.6***
V3.5	Did the baby have difficulty breathing?	1. Yes<br>2. No<br>9. Don't know	☐
V3.6	Was anything done to try to help the baby breathe at birth?	1. Yes<br>2. No<br>9. Don't know	☐
V3.7	Did the baby cry immediately after birth?	1. Yes<br>2. No<br>9. Don't know	☐ ***1 → VQ3.9***
V3.8	How long after birth did the baby first cry?<br><br>*[Mark ONE response]*	1. Within 5 minutes<br>2. Within 6-30 minutes<br>3. More than 30 minutes<br>4. Never<br>9. Don't know	☐ ***4 → SQ5a.4***
V3.9	Did the baby stop being able to cry?	1. Yes<br>2. No<br>9. Don't know	☐ ***2 or 9 → SQ5a.4***

V3.10	How long before the baby died did the baby stop crying?	1. Less than one day<br>2. One day or more<br>9. Don't know	☐
S5a.4	How long after birth was the baby first bathed?	1. Less than 1 hour<br>2. 1-23 hours<br>3. 24-72 hours (1-3 days)<br>4. More than 72 hours (3 days)<br>5. Not bathed<br>9. Don't know	☐
S5a.5	Was anything done to keep the baby warm on the first day after birth?	1. Yes<br>2. No<br>9. Don't know	☐ ***2 or 9 → SQ5a.6***
S5a.5.1	What was done?<br><br>*[Multiple answers allowed.]*<br><br>*For each mentioned, ask:*<br>How soon after birth was this done?	1. Dried/wiped ....<br>2. Wrapped in a blanket ....<br>3. Skin-to-skin contact ....<br>4. Incubator ....<br>5. Other ....<br><br>*(specify other)*	Done | How soon after birth (<u><1hr</u> <u><6</u> <u>6-24</u> <u>>24</u> <u>DK</u>)<br>1. ☐ ... | 1.☐ 2.☐ 3.☐ 4.☐ 9.☐<br>2. ☐ ... | 1.☐ 2.☐ 3.☐ 4.☐ 8.☐<br>3. ☐ ... | 1.☐ 2.☐ 3.☐ 4.☐ 9.☐<br>4. ☐ ... | 1.☐ 2.☐ 3.☐ 4.☐ 9.☐<br>5. ☐ ... | 1.☐ 2.☐ 3.☐ 4.☐ 9.☐<br><br>________________
S5a.6	Did (you / the mother) or a wet nurse ever breastfeed the baby?	1. Yes<br>2. No<br>9. Don't know	☐ ***2 or 9 → SQ5a.7***
S5a.6.1	How long after birth was the baby first put to the breast?<br><br>*[If immediately or less than 1 hour, record '00' hours.]*<br>*[If less than 24 hours, record hours; otherwise record days.]*		___ ___ Days<br>*(DK = 99)*<br>OR<br>___ ___ Hours<br>*(DK = 99)*
S5a.6.2	Was the baby being breastfed at the time when the fatal illness began?	1. Yes<br>2. No<br>9. Don't know	☐
S5a.7	At the time the fatal illness began, was the baby being given any other liquid, including non-human milk or formula, fruit juice, tea or water, or any semisolid or soft foods such as cereal?<br><br>*[Multiple answers allowed. Probe, and record all liquids and foods given.]*	1. Non-human milk or pre-mixed formula ..<br>2. Powdered formula mixed with a liquid ...<br>3. Juice, water and/or water-based drinks .<br>4. ORS ....<br>5. Drops or syrups (vitamins, medicines)<br>6. Semi-solid or soft foods ....<br>7. Nothing else, <u>only</u> given breast milk ......<br>9. Don't know..........	1. ☐<br>2. ☐<br>3. ☐<br>4. ☐<br>5. ☐<br>6. ☐<br>7. ☐<br>9. ☐
V3.11	Was the baby able to suckle in a normal way during the first day of life?	1. Yes<br>2. No<br>9. Don't know	☐ ***1 → VQ3.13***
V3.12	Did the baby ever suckle in a normal way?	1. Yes<br>2. No<br>9. Don't know	☐ ***2 or 9 → VQ3.17***
V3.13	Did the baby stop being able to suckle in a normal way?	1. Yes<br>2. No<br>9. Don't know	☐ ***2 or 9 → VQ3.17***
V3.14	How long after birth did the baby stop suckling?<br><br>*[Less than 24 hours = "00" days]*		___ ___ Days<br>*(DK = 99)*
V3.15	How long before s/he died did the baby stop suckling?	1. Less than one day<br>2. One day or more<br>9. Don't know	☐

V3.16	Was the baby able to open her/his mouth at the time s/he stopped suckling?	1. Yes<br>2. No<br>9. Don't know	☐
V3.17	During the illness that led to death, did the baby have difficult breathing?	1. Yes<br>2. No<br>9. Don't know	☐ ***2 or 9 → VQ3.20***
V3.18	At what age did the difficult breathing start?<br><br>*[Less than 24 hours = "00" days]*		___ ___ Days<br>*(DK = 99)*
V3.19	For how many days did the difficult breathing last?<br><br>*[Less than 24 hours = "00" days]*		___ ___ Days<br>*(DK = 99)*
V3.20	During the illness that led to death, did the baby have fast breathing?	1. Yes<br>2. No<br>9. Don't know	☐ ***2 or 9 → VQ3.23***
V3.21	At what age did the fast breathing start?<br><br>*[Less than 24 hours = "00" days]*		___ ___ Days<br>*(DK = 99)*
V3.22	For how many days did the fast breathing last?<br><br>*[Less than 24 hours = "00" days]*		___ ___ Days<br>*(DK = 99)*
V3.23	During the illness that led to death, did the baby have indrawing of the chest?<br><br>*[Show photo]*	1. Yes<br>2. No<br>9. Don't know	☐
V3.24	During the illness that led to death, did the baby have grunting?<br><br>*[Demonstrate grunting]*	1. Yes<br>2. No<br>9. Don't know	☐
V3.25	During the illness that led to death, did the baby have spasms or convulsions?	1. Yes<br>2. No<br>9. Don't know	☐
V3.26	During the illness that led to death, did the baby have fever?	1. Yes<br>2. No<br>9. Don't know	☐ ***2 or 9 → VQ3.29***
V3.27	At what age did the fever start?<br><br>*[Less than 24 hours = "00" days]*		___ ___ Days<br>*(DK = 99)*
V3.28	How many days did the fever last?<br><br>*[Less than 24 hours = "00" days]*		___ ___ Days<br>*(DK = 99)*
V3.29	During the illness that led to death, did the baby become cold to touch?	1. Yes<br>2. No<br>9. Don't know	☐ ***2 or 9 → VQ3.32***
V3.30	At what age did the baby start feeling cold to touch?<br><br>*[Less than 24 hours = "00" days]*		___ ___ Days<br>*(DK = 99)*
V3.31	How many days did the baby feel cold to touch?<br><br>*[Less than 24 hours = "00" days]*		___ ___ Days<br>*(DK = 99)*
V3.32	During the illness that led to death, did the baby become lethargic, after a period of normal activity?	1. Yes<br>2. No<br>9. Don't know	☐
V3.33	During the illness that led to death, did the baby become unresponsive or unconscious?	1. Yes<br>2. No<br>9. Don't know	☐

V3.34	During the illness that led to death, did the baby have a bulging fontanelle?<br><br>*[Show photo]*	1. Yes<br>2. No<br>9. Don't know	☐
V3.35	During the illness that led to death, did the baby have pus drainage from the umbilical cord stump?	1. Yes<br>2. No<br>9. Don't know	☐
V3.36	During the illness that led to death, did the baby have redness of the umbilical cord stump?	1. Yes<br>2. No<br>9. Don't know	☐ ***2 or 9 → VQ3.38***
V3.37	Did the redness of the umbilical cord stump extend onto the abdominal skin?	1. Yes<br>2. No<br>9. Don't know	☐
V3.38	During the illness that led to death, did the baby have skin bumps containing pus or a single large area with pus?	1. Yes<br>2. No<br>9. Don't know	☐
V3.39	During the illness that led to death, did the baby have ulcer(s) (pits)?	1. Yes<br>2. No<br>9. Don't know	☐
V3.40	During the illness that led to death, did the baby have an area(s) of skin with redness and swelling?	1. Yes<br>2. No<br>9. Don't know	☐
V3.41	During the illness that led to death, did s/he have areas of the skin that turned black?	1. Yes<br>2. No<br>9. Don't know	☐
V3.42	During the illness that led to death, did the baby bleed from anywhere?	1. Yes<br>2. No<br>9. Don't know	☐ ***2 or 9 → VQ3.44***
V3.43	*Record from where did the baby bleed:*		
V3.44	During the illness that led to death, did s/he have more frequent loose or liquid stools than usual?	1. Yes<br>2. No<br>9. Don't know	☐ ***2 or 9 → VQ3.46***
V3.45	How many stools did the baby have on the day that diarrhea/loose liquid stools were most frequent?		___ ___ Stools<br>*(DK = 99)*
V3.46	During the illness that led to death, did s/he vomit everything?	1. Yes<br>2. No<br>9. Don't know	☐
V3.47	During the illness that led to death, did s/he have yellow skin?	1. Yes<br>2. No<br>9. Don't know	☐
V3.48	During the illness that led to death, did the baby have yellow eyes?	1. Yes<br>2. No<br>9. Don't know	☐
V3.49	Did the infant appear to be healthy and then just die suddenly?	1. Yes<br>2. No<br>9. Don't know	☐
S5a.8	*Check SQ4.17 to determine if the baby was born in a health facility (codes 1-2):*	1. Yes, born in a health facility<br>2. Not born in a health facility<br>9. Don't know	☐ ***2 or 9 → SQ5a.10***
S5a.8.1	Did the baby leave the delivery facility alive or did s/he die in the facility?	1. Yes, left alive<br>2. Died in the facility<br>9. Don't know	☐ ***2 or 9 → SQ6.1***

S5a.8.2	How soon after birth did the baby leave the facility?<br><br>*[Record hours if less than 24 hours—if less than 1 hour, record '00' hours; Record days if 1 day or more.]*		___ ___ Days<br>*(DK = 99)*<br>OR<br>___ ___ Hours<br>*(DK = 99)*
S5a.8.3	Was the child examined by a health worker prior to discharge?	1. Yes<br>2. No<br>9. Don't know	☐
S5a.9	Did (you / the mother) receive any counselling by a health worker prior to discharge?	1. Yes<br>2. No<br>9. Don't know	☐ ***2 or 9 → SQ5a.10***
S5a.9.1	What (were you / was she) counselled on?<br><br>*[Multiple answers allowed].*<br><br>*Probe:* Anything else?	1. Breastfeeding<br>2. Immunization<br>3. Post-natal care attendance<br>4. Danger signs of newborn illness<br>5. Other (specify)<br>9. Don't know	1. ☐<br>2. ☐<br>3. ☐<br>4. ☐<br>5. ☐ ____________<br>9. ☐
S5a.10	Was the baby ever seen by a health worker or nurse at home or in the community, or by a doctor or nurse at a health facility <u>before</u> the fatal illness began?<br><br>*[Multiple answers allowed.]*<br><br>*For each mentioned, ask:*<br>How many times was the baby seen by a <PROVIDER TYPE at PLACE> before the fatal illness began?<br><br>*Then ask:*<br>When was the baby first seen by (this / <u>any</u> of these) provider(s)?	1. CHW or nurse at home/in community....<br><br>2. Doctor or nurse at a health facility.........<br>3. Never seen.........<br>9. Don't know.........	<u>Seen</u> / <u>Times</u> / <u>First visit</u><br>1. ☐... ..... ___ ___<br>2. ☐... ..... ___ ___<br>3. ☐<br>9. ☐<br>First visit: ___ ___ Days old *(<1 = 00; DK = 99)*
S5a.11	<u>Before</u> the fatal illness began, did <NAME> suffer from any of the following known conditions:<br><br>*[Read out all conditions and check "Yes," "No" or "Don't know" for each.]*<br><br>*If "Yes," then ask:* Was s/he provided any treatment for this condition?	1. Preterm birth.........<br>a. Was s/he given special nutrition?....<br>b. Was s/he given "kangaroo care"?....<br>2. Malformation (from the time of birth):<br>a. Head, neck and/or back ....<br>b. Mouth/palate ....<br>c. Heart ....<br>d. Arms and/or legs ....<br>3. Other ....<br><br>*(specify other)*.........	<u>Suffered from</u> (<u>Yes</u> <u>No</u> <u>DK</u>) / <u>Treatment</u> (<u>Yes</u> <u>No</u> <u>DK</u>)<br>1. Preterm birth: 1. ☐ 2. ☐ 9. ☐ / —<br>a. .... / 1. ☐ 2. ☐ 9. ☐<br>b. .... / 1. ☐ 2. ☐ 9. ☐<br>2a. 1. ☐ 2. ☐ 9. ☐ / 1. ☐ 2. ☐ 9. ☐<br>2b. 1. ☐ 2. ☐ 9. ☐ / 1. ☐ 2. ☐ 9. ☐<br>2c. 1. ☐ 2. ☐ 9. ☐ / 1. ☐ 2. ☐ 9. ☐<br>2d. 1. ☐ 2. ☐ 9. ☐ / 1. ☐ 2. ☐ 9. ☐<br>3. 1. ☐ 2. ☐ 9. ☐ / 1. ☐ 2. ☐ 9. ☐<br>____________

Inst_1: STOP – If VQ1.26 = 1 (Neonatal death) → SQ6.1

SA Module 5b: Preventive care of post-neonates (FOR CHILD DEATHS 28 DAYS—59 MONTHS OLD)

Read: Now I would like to ask you about the care of the child before the fatal illness began.

S5b.1	Where (do you / does the mother) cook?	1. Inside the house<br>2. Outside the house<br>3. In a structure outside the house<br>9. Don’t know	☐
S5b.2	When (you / the mother) cooked, was <NAME> usually beside or carried by (you / her)?	1. Yes<br>2. No<br>9. Don’t know	☐
S5b.3	*Skip SQ5b.3 in areas wo/malaria.*<br><br>Before (her / his) fatal illness began, did <NAME> sleep under an insecticide treated bednet?	1. Yes, usually or always<br>2. Yes, sometimes<br>3. Never<br>9. Don’t know	☐
S5b.4	Did (you / the mother) or a wet nurse ever breastfeed <NAME>?	3. Yes<br>4. No<br>9. Don’t know	☐ ***2 or 9 → SQ5b.5***
S5b.4.1	Was <NAME> being breastfed at the time (her / his) fatal illness began?	1. Yes<br>2. No<br>9. Don’t know	☐ ***1 or 9 → SQ5b.5***
S5b.4.2	How old was <NAME> when s/he was last breastfed?		___ ___ Months<br>*(<1 = 00; DK = 99)*
S5b.5	At the time the fatal illness began, was <NAME> being given any other liquid, including non-human milk or formula, fruit juice, tea or water, or any solid, semisolid, or soft foods?<br><br>*[Multiple answers allowed. Probe, and record all liquids and foods given.]*	1. Non-human milk or pre-mixed formula ..<br>2. Powdered formula mixed with a liquid ...<br>3. Juice, water and/or water-based drinks .<br>4. ORS ....<br>5. Drops or syrups (vitamins, medicines)...<br>6. Solid, semi-solid or soft foods...............<br>7. Nothing else, <u>only</u> given breast milk ......<br>9. Don’t know..	1. ☐<br>2. ☐<br>3. ☐<br>4. ☐<br>5. ☐ (1–5) → ***SQ5b.6***<br>6. ☐<br>7. ☐<br>9. ☐ (7, 9) → ***SQ5b.6***
S5b.5.1	On most days <u>before</u> the illness began, how many <u>times</u> did <NAME> eat solid, semisolid, or soft foods other than liquids during the day or night?		___ ___ Times<br>*(DK = 99)*
S5b.5.2	Which of the following food types did <NAME> typically eat <u>every</u> day?<br><br>*[Read out all options and check “Yes,” “No” or “Don’t know” for each.]*	1. Grains, roots and tubers.......................<br>2. Legumes and nuts...............................<br>3. Dairy products (milk, yogurt, cheese) ....<br>4. Flesh foods (meat, fish, poultry, organs)<br>5. Eggs ....<br>6. Vitamin-A rich fruits and vegetables ......<br>7. Other fruits and vegetables	<u>Yes</u> <u>No</u> <u>DK</u><br>1. ☐ 2. ☐ 9. ☐<br>1. ☐ 2. ☐ 9. ☐<br>1. ☐ 2. ☐ 9. ☐<br>1. ☐ 2. ☐ 9. ☐<br>1. ☐ 2. ☐ 9. ☐<br>1. ☐ 2. ☐ 9. ☐<br>1. ☐ 2. ☐ 9. ☐
S5b.6	Did <NAME> drink any liquids or semi-solid foods from a bottle with a nipple or teat?	1. Yes<br>2. No<br>9. Don’t know	☐
S5b.7	Now I would like to ask about the chlid’s vaccinations. Do you have a card where <NAME>’s vaccinations are written down?<br><br>*If “Yes,” ask:* May I see it please?	1. Yes, seen<br>2. Yes, but not seen<br>3. No card	☐ ***2 or 3 → SQ5b.8***

S5b.7.1	Did <NAME> receive any vaccinations that are not included on this card, including vaccinations received in a national immunization day campaign?<br><br>*If "Yes," probe for vaccinations received but not recorded on the card.*<br><br>*[Record 'Yes' only if BCG, Polio 0-3, Hepatitis B1-3, DPT 1-3, PENTA 1-3, Measles or Yellow Fever vaccine(s) mentioned.]*	1. Yes (received BCG, Polio 0-3, DPT 1-3 or PENTA 1-3, Measles, Yellow Fever and/or Hep B1-3 vaccinations that are not recorded on the card)<br>2. No<br>9. Don't know	☐ ***1 → Write '66' in the corresponding day column below for each vaccination received but not recorded on the card.***
	Copy vaccination date for each vaccine from the card. Record "99" or "9999" for partially unknown dates.<br><br>*Write '88' in 'day' column if card shows that a vaccination was given, but no date is recorded.*<br><br>*Do not leave any rows blank. Record "00" in the 'day' column for each vaccination that was not given.*	(see vaccination date grid below)	

Vaccine	Day		Month		Year				
BCG									BCG
POLIO 0 (given at birth)..									P0
POLIO 1									P1
POLIO 2									P2
POLIO 3									P3
Hepatitis B1									HEP1
Hepatitis B2									HEP2
Hepatitis B3									HEP3
DPT 1									DPT1
DPT 2									DPT2
DPT 3									DPT3
PENTA1									PNT1
PENTA2									PNT2
PENTA3									PNT3
MEASLES									MSL
YELLOW FEVER									YF

S5b.8	Did <NAME> ever receive any vaccinations to prevent her/him from getting diseases, including vaccinations received in a national immunization day campaign?	1. Yes<br>2. No<br>9. Don't know	☐ ***2 or 9 → SQ5b.10***
	Please tell me if <NAME> received any of the following vaccinations:		
.1	A BCG vaccination against tuberculosis, that is, an injection in the arm or shoulder that usually causes a scar?	1. Yes<br>2. No<br>9. Don't know	☐
.2	Polio vaccine, that is, drops in the mouth?	1. Yes<br>2. No<br>9. Don't know	☐ ***2 or 9 → SQ5b.8.5***
.3	When was the first polio vaccine received, just after birth or later?	1. Just after birth<br>2. Later<br>9. Don't know	☐
.4	How many times was the polio vaccine received?		___ ___ Times<br>*(DK = 99)*
.5	A Hep B vaccination, that is, an injection in the right thigh, sometimes given just after birth or at the same time as polio drops?	1. Yes<br>2. No<br>9. Don't know	☐ ***2 or 9 → SQ5b.8.7***

.6	How many times was a Hep B vaccination received?		___ ___ Times<br>*(DK = 99)*
.7	A DPT vaccination, that is, an injection in the thighs or buttocks, sometimes given at the same time as polio drops or a Hep B vaccination?	1. Yes<br>2. No<br>9. Don't know	☐ ***2 or 9 → SQ5b.8.9***
.8	How many times was a DPT vaccination received?		___ ___ Times<br>*(DK = 99)*
.9	A PENTA vaccination, that is, an injection in the thighs or buttocks instead of a Hep B or DPT vaccination, sometimes given at the same time as polio drops?	1. Yes<br>2. No<br>9. Don't know	☐ ***2 or 9 → SQ5b.8.11***
.10	How many times was a PENTA vaccination received?		___ ___ Times<br>*(DK = 99)*
.11	A measles or MMR injection, that is, a shot in the arm at the age of 9 months or older, to prevent measles?	1. Yes<br>2. No<br>9. Don't know	☐
.12	A yellow fever vaccination, that is, an injection given in the arm after the child is 9 months old?	1. Yes<br>2. No<br>9. Don't know	☐
S5b.9	Were any of the vaccinations <NAME> received given as part of a national immunization day campaign?	1. Yes<br>2. No<br>9. Don't know	☐ ***2 or 9 → SQ5b.10***
S5b.9.1	At which national immunization day campaigns did <NAME> receive vaccinations?	1. National immunization campaign	1. ☐
S5b.10	(Before / In the six months before) the fatal illness, did <NAME> receive one or more vitamin A doses like this?<br><br>*[Read "Before…" if the child lived less than 6 months.]*<br><br>*[Show ampoule/capsule/syrup]*	1. Yes, 1 dose<br>2. Yes, 2 or more doses<br>3. No<br>9. Don't know	☐
S5b.11	<u>Before</u> the fatal illness began, did <NAME> suffer from any of the following known conditions:<br><br>*[Read out all conditions and check "Yes," "No" or "Don't know" for each.]*<br><br>*If "Yes," then ask:* Was s/he provided any treatment for this condition?		

	Suffered from: Yes	No	DK	Treatment: Yes	No	DK
1. Low height or weight (malnutrition)	1. ☐	2. ☐	9. ☐	1. ☐	2. ☐	9. ☐
2. Malformation (from the time of birth):						
a. Head, neck and/or back	1. ☐	2. ☐	9. ☐	1. ☐	2. ☐	9. ☐
b. Mouth/palate	1. ☐	2. ☐	9. ☐	1. ☐	2. ☐	9. ☐
c. Heart	1. ☐	2. ☐	9. ☐	1. ☐	2. ☐	9. ☐
d. Arms and/or legs	1. ☐	2. ☐	9. ☐	1. ☐	2. ☐	9. ☐
3. Asthma	1. ☐	2. ☐	9. ☐	1. ☐	2. ☐	9. ☐
4. Heart disease	1. ☐	2. ☐	9. ☐	1. ☐	2. ☐	9. ☐
5. Tuberculosis	1. ☐	2. ☐	9. ☐	1. ☐	2. ☐	9. ☐
6. Epilepsy/convulsion	1. ☐	2. ☐	9. ☐	1. ☐	2. ☐	9. ☐
7. Other	1. ☐	2. ☐	9. ☐	1. ☐	2. ☐	9. ☐
(specify other)	____________________					

<u>VA Section 4: Infant and child deaths (FOR CHILD DEATHS 28 DAYS—59 MONTHS OLD)</u>

Read: Now I'd like to ask you about <NAME>'s illness.

V4.1	During the illness that led to death, did the <NAME> have a fever?	1. Yes<br>2. No<br>9. Don't know	☐ ***2 or 9 → VQ4.6***

V4.2	How many days did the fever last?<br><br>*[Less than 24 hours = "00" days]*		___ ___ Days<br>*(DK = 99)*
V4.3	Did the fever continue until death?	1. Yes<br>2. No<br>9. Don't know	☐ ***2 or 9 → VQ4.6***
V4.4	How severe was the fever?	1. Mild<br>2. Moderate<br>3. Severe<br>9. Don't know	☐
V4.5	What was the pattern of the fever?	1. Continuous<br>2. On and off<br>3. Only at night<br>9. Don't know	☐
V4.6	During the illness that led to death, did <NAME> have more frequent loose or liquid stools than usual?	1. Yes<br>2. No<br>9. Don't know	☐ ***2 or 9 → VQ4.12***
V4.7	How many stools did <NAME> have on the day that loose liquid stools were most frequent?		___ ___ Stools<br>*(DK = 99)*
V4.8	How many days before death did the frequent loose or liquid stools start?<br><br>*[Less than 24 hours = "00" days]*		___ ___ Days<br>*(DK = 99)*
V4.9	Did the frequent loose or liquid stools continue until death?	1. Yes<br>2. No<br>9. Don't know	☐ ***1 or 9 → VQ4.11***
V4.10	How many days before death did the loose or liquid stools stop?<br><br>*[Less than 24 hours = "00" days]*		___ ___ Days<br>*(DK = 99)*
V4.11	Was there visible blood in the loose or liquid stools?	1. Yes<br>2. No<br>9. Don't know	☐
V4.12	During the illness that led to death, did the child have a cough?	1. Yes<br>2. No<br>9. Don't know	☐ ***2 or 9 → VQ4.16***
V4.13	For how many days did the cough last?<br><br>*[Less than 24 hours = "00" days]*		___ ___ Days<br>*(DK = 99)*
V4.14	Was the cough very severe?	1. Yes<br>2. No<br>9. Don't know	☐
V4.15	Did the child vomit after s/he coughed?	1. Yes<br>2. No<br>9. Don't know	☐
V4.16	During the illness that led to death, did <NAME> have difficult breathing?	1. Yes<br>2. No<br>9. Don't know	☐ ***2 or 9 → VQ4.18***
V4.17	For how many days did the difficult breathing last?<br><br>*[Less than 24 hours = "00" days]*		___ ___ Days<br>*(DK = 99)*
V4.18	During the illness that led to death, did <NAME> have fast breathing?	1. Yes<br>2. No<br>9. Don't know	☐ ***2 or 9 → VQ4.20***

Inst_1: If both VQ4.16 and VQ4.18 = 2 or 9 → VQ4.25			
V4.19	For how many days did the fast breathing last?<br>*[Less than 24 hours = "00" days]*		___ ___ Days<br>*(DK = 99)*
V4.20	During the illness that led to death, did s/he have indrawing of the chest?	1. Yes<br>2. No<br>9. Don't know	☐
V4.21	During the illness that led to death, did her/his breathing sound like any of the following?<br>*[Demonstrate each sound]*		
V4.22	Stridor	1. Yes<br>2. No<br>9. Don't know	☐
V4.23	Grunting	1. Yes<br>2. No<br>9. Don't know	☐
V4.24	Wheezing	1. Yes<br>2. No<br>9. Don't know	☐
V4.25	Did <NAME> experience any generalized convulsions or fits during the illness that led to death?	1. Yes<br>2. No<br>9. Don't know	☐
V4.26	Was <NAME> unconscious during the illness that led to death?	1. Yes<br>2. No<br>9. Don't know	☐ ***2 or 9 → VQ4.28***
V4.27	How long before death did unconsciousness start?	1. Less than 6 hours<br>2. 6-23 hours<br>3. 24 hours or more<br>9. Don't know	☐
V4.28	Did <NAME> have a stiff neck during the illness that led to death?<br>*[Demonstrate]*	1. Yes<br>2. No<br>9. Don't know	☐
V4.29	Did <NAME> have a bulging fontanelle during the illness that led to death?<br>*[Show photo]*	1. Yes<br>2. No<br>9. Don't know	☐
V4.30	During the month before s/he died, did <NAME> have a skin rash?	1. Yes<br>2. No<br>9. Don't know	☐ ***2 or 9 → VQ4.35***
V4.31	Where was the rash?	1. Face<br>2. Trunk/Abdomen<br>3. Extremities<br>4. Everywhere<br>9. Don't know	☐
V4.32	Where did the rash start?	1. Face<br>2. Trunk/Abdomen<br>3. Extremities<br>4. Everywhere<br>9. Don't know	☐

V4.33	How many days did the rash last?		___ ___ Days<br>*(DK = 99)*
V4.34	Did the rash have blisters containing clear fluid?	1. Yes<br>2. No<br>9. Don't know	☐
V4.35	During the illness that led to death, did <NAME>'s limbs (legs, arms) become very thin?<br><br>*[Show photo]*	1. Yes<br>2. No<br>9. Don't know	☐
V4.36	During the illness that led to death, did <NAME> have swollen legs or feet?	1. Yes<br>2. No<br>9. Don't know	☐ ***2 or 9 → VQ4.38***
V4.37	How long did the swelling last?<br><br>*[Record days or weeks.]*		___ ___ Days<br>*(DK = 99)*<br><br>___ ___ Weeks<br>*(DK = 99)*
V4.38	During the illness that led to death, did <NAME>'s skin flake off in patches?	1. Yes<br>2. No<br>9. Don't know	☐
V4.39	Did <NAME>'s hair change in color to a reddish or yellowish color?	1. Yes<br>2. No<br>9. Don't know	☐
V4.40	Did <NAME> have a protruding belly?	1. Yes<br>2. No<br>9. Don't know	☐
V4.41	During the illness that led to death, did <NAME> suffer from "lack of blood" or "pallor"?	1. Yes<br>2. No<br>9. Don't know	☐
V4.42	During the illness that led to death, did <NAME> have swelling in the armpits?	1. Yes<br>2. No<br>9. Don't know	☐
V4.43	During the illness that led to death, did <NAME> have a whitish rash inside the mouth or on the tongue?	1. Yes<br>2. No<br>9. Don't know	☐
V4.44	During the illness that led to death, did <NAME> bleed from anywhere?	1. Yes<br>2. No<br>9. Don't know	☐ ***2 or 9 → VQ4.46***
V4.45	*Record from where s/he bled:*		
V4.46	During the illness that led to death, did s/he have areas of the skin that turned black?	1. Yes<br>2. No<br>8. Don't know	☐
V4.47	Did <NAME> suffer from an injury or accident such as…?<br><br>*[Ask the respondent each in sequence and mark each as "Yes," "No" or "Don't know."]*	1. a road traffic crash/injury?....................<br>2. a fall?..<br>3. drowning?...<br>4. poisoning?..<br>Did s/he suffer:<br>5. a bite or sting by a venomous animal?..<br>6. a burn?...<br>7. from violence (homicide, abuse)?..........	Yes No DK<br>1. ☐ 2. ☐ 9. ☐<br>1. ☐ 2. ☐ 9. ☐<br>1. ☐ 2. ☐ 9. ☐<br>1. ☐ 2. ☐ 9. ☐<br>1. ☐ 2. ☐ 9. ☐<br>1. ☐ 2. ☐ 9. ☐<br>1. ☐ 2. ☐ 9. ☐<br>1. ☐ 2. ☐ 9. ☐<br>***All = 2 or 9 → SQ6.1***

		8. any other injury?...................................<br><br>*(If "Yes," then specify)*..........................	___________________________
V4.48	Was the injury or accident intentionally inflicted by someone else?	1. Yes<br>2. No<br>8. Don't know	☐
V4.49	How long did <NAME> survive after the injury or accident?<br><br>*[Record hours if less than 24 hours—Less than 1 hour = "00" hours; Record days if 1 day or more.]*		___ ___ Hours<br>*(DK = 99)*
			___ ___ Days<br>*(DK = 99)*

SA Module 6: Care-seeking for the child's fatal illness (FOR NN & CHILD DEATHS 0—59 MONTHS OLD)

Read: Now, I'd like to ask you about <NAME>'s fatal illness and the care and treatments that s/he received.

S6.1	Who first noticed that <NAME> was ill?	1. The respondent<br>2. Other relative, neighbor, friend<br>3. CHW or nurse at home or in community<br>4. Doctor or nurse at a health facility<br>5. Other *(specify)*......................................	☐<br>______________________
S6.2	Earlier you said that <NAME> had <SYMPTOM(S)> during her/his illness. *[Read back all the child's symptoms from VA section 3 (for neonates) or VA section 4 (for children).]*<br><br>How did <SQ6.1 PERSON> first know that <NAME> was ill? Which of these symptoms did s/he have at that time?<br><br>What symptoms did s/he have next? On what day of the illness did these symptoms start?<br><br>*[Probe until all the symptoms are recorded in the order they appeared.]*	**Symptoms in order of appearance**<br>1.<br>2.<br>3.<br>4.<br>5.<br>6.<br>7.	**Illness day the symptom started**
S6.3	When <SQ6.1 PERSON> first noticed that <NAME> was ill, was s/he…<br><br>*[Read the choices for each condition.]*	1. Feeding normally, poorly, or not at all....<br>2. Alert, drowsy, or unconscious...............<br>3. Normally active, less active than normal, or not moving..........................	Normal / Medium / Abnormal / DK<br>1. ☐ 2. ☐ 3. ☐ 9. ☐<br>1. ☐ 2. ☐ 3. ☐ 9. ☐<br>1. ☐ 2. ☐ 3. ☐ 9. ☐
S6.4	Did <NAME> receive, or did you seek or try to seek, any care or treatment for the fatal illness?	1. Yes<br>2. No—care not needed, given or sought<br>3. No— died immediately<br>9. Don't know	☐ ***2 → SQ6.6***<br>***3 or 9 → VQ5.10***

S6.5 Please tell me everything you did for <NAME>'s fatal illness inside the home and all the places outside the home you took or tried to take (her / him) for health care. Start with the first care or treatment <NAME> received and then, in order, tell me all the other care and treatments s/he received. Also tell me when and for what symptoms you took each action.

[Include any provider <NAME> did not reach because s/he died before leaving home or on route.]

(1) Check one other care or health provider box for each action row. (2) For neonatal deaths only: If the illness began at the health provider where the child was delivered, then mark that as Action 1 and check the "illness began at provider" box. (3) Record the illness day each action was taken. (4) Ensure no action was taken for a symptom before it started (in SQ6.2).

	**(1) Other care**			**(1) Health Providers**						
**Action #**	**Home care (own, relative, neigh-bor, friend)**	**Tradi-tional or non-formal provider**	**Phar-macist or drug seller**	**Trained CH Worker, nurse, or midwife**	**Private doctor (formal/ unsure)**	**NGO or govt. clinic**	**Hospital**	**(2) Illness began at provider where child was delivered**	**(3) Illness day the action was taken**	**(4) For what symptom(s) was the action taken?**
1.	☐	☐	☐	☐	☐	☐	☐	☐	___ ___ *(DK = 99)*	
2.	☐	☐	☐	☐	☐	☐	☐		___ ___ *(DK = 99)*	
3.	☐	☐	☐	☐	☐	☐	☐		___ ___ *(DK = 99)*	

4.	☐	☐	☐	☐	☐	☐	☐		___ ___ (DK = 99)	
5.	☐	☐	☐	☐	☐	☐	☐		___ ___ (DK = 99)	
6.	☐	☐	☐	☐	☐	☐	☐		___ ___ (DK = 99)	
7.	☐	☐	☐	☐	☐	☐	☐		___ ___ (DK = 99)	

Inst_1: (For neonatal deaths only) If illness began at health provider where child was delivered: And did not fill L&D matrix (module 4) → SQ6.10; And filled L&D matrix (module 4) → SQ6.16

S6.6	*If no care given or sought, ask:* Who decided that <NAME> did not need any care or treatment for the illness?<br><br>*If any care given or sought, ask:* Who decided that <ACTION 1> was the first thing to do for <NAME>'s illness?<br><br>*[Record the one main decision maker.]*	1. Child's mother<br>2. Child's father<br>3. Child's aunt<br>4. Child's uncle<br>5. Child's grandmother<br>6. Child's paternal grandfather<br>7. Child's maternal grandfather<br>8. Other *(specify)*<br>9. Don't know	1. ☐<br>2. ☐<br>3. ☐<br>4. ☐<br>5. ☐<br>6. ☐<br>7. ☐<br>8. ☐ ____________<br>9. ☐
S6.7	*If never taken to a health provider, ask:* Did you have any concerns or problems that kept you from taking <NAME> to a health provider during his/her illness?<br><br>*If taken to a health provider, ask:* Did you have to overcome any concerns or problems to take <NAME> to the (first) health provider?	1. Yes<br>2. No<br>9. Don't know	☐ ***2 or 9 → Inst_2***
S6.7.1	What concerns or problems did you have?<br><br>*Prompt:* Was there anything else?<br><br>*[Multiple answers allowed.]*	1. Did not think child was sick enough to need health care<br>2. No one available to go with caregiver<br>3. Too much time from her regular duties<br>4. Someone else *(specify)* had to decide<br>5. Too far to travel<br>6. No transportation available<br>7. Cost (transport, health care, other)<br>8. Not satisfied with available health care<br>9. Problem required traditional care<br>10. Thought child was too sick to travel<br>11. Thought child will die no matter what<br>12. Was late at night (transportation or provider not available)<br>13. Other *(specify)*<br>99. Don't know	1. ☐<br>2. ☐<br>3. ☐<br>4. ☐ ____________<br>5. ☐<br>6. ☐<br>7. ☐<br>8. ☐<br>9. ☐<br>10. ☐<br>11. ☐<br>12. ☐<br>13. ☐ ____________<br>99. ☐

Inst_2: If SQ6.4 = 2 (No care given) or If SQ6.5 ≠ "Health Provider" (Never took and never tried to take to a health provider) → SQ6.39

S6.8	*Refer to SQ6.5 for the first health provider and related symptoms:*<br>You mentioned that you took <NAME> to the (first) health provider, I mean the <FIRST HEALTH PROVIDER> for <SYMPTOM(S)>. How long had <NAME> had (this / these) symptom(s) when it was decided to take him/her to the <FIRST HEALTH PROVIDER>?<br><br>*[Read "…to the first…" if took or tried to take to more than one health provider.]*<br><br>*[Mark days, hours &/or minutes as needed: e.g. 00 day, 02 hours, 10 minutes]*	___ ___ Days *(DK = 99)*<br>___ ___ Hours *(DK = 99)*<br>___ ___ Minutes *(DK = 99)*

Child illness matrix instructions: *Ask the following questions for the first and last health providers where care was sought or tried to be sought for the fatal illness. Ask all the questions for the first provider before going on to the last.*

Before asking about the first health provider, read:
Now I would like to ask you about your visit to the (first) health provider. *[Read "first" if went or tried to go to more than one provider.]*

Before asking about the last health provider, read:
Now I would like to ask you about your visit to the last health provider, I mean the <LAST HEALTH PROVIDER>.

**– CHILD ILLNESS MATRIX QUESTIONS –**		**FIRST HEALTH PROVIDER**	**LAST HEALTH PROVIDER**
At the time when it was decided to take <NAME> to the <FIRST/LAST HEALTH PROVIDER>, was s/he…<br><br>*[Read the choices for each condition.]*	1. Feeding normally, poorly, or not at all ....<br>2. Alert, drowsy, or unconscious ......<br>3. Normally active, less active than normal, or not moving	S6.9<br>Nrml Med Abnrm DK<br>1. ☐ 2. ☐ 3. ☐ 9. ☐<br>1. ☐ 2. ☐ 3. ☐ 9. ☐<br>1. ☐ 2. ☐ 3. ☐ 9. ☐	S6.24<br>Nrml Med Abnrm DK<br>1. ☐ 2. ☐ 3. ☐ 9. ☐<br>1. ☐ 2. ☐ 3. ☐ 9. ☐<br>1. ☐ 2. ☐ 3. ☐ 9. ☐
What was the name of the <FIRST/LAST HEALTH PROVIDER> where you took <NAME>?<br><br>*Probe to identify the type of provider.*	1. Hospital (Government)<br>2. Hospital (NGO)<br>3. Hospital (Private)<br>4. Health center (Government)<br>5. Health center (NGO)<br>6. Health post (Government)<br>7. Health post (NGO)<br>8. Private doctor/clinic (Formal)<br>9. Private doctor/clinic (?Formal?)<br>10. Trained community health worker, nurse, or midwife<br>99. Don't know	S6.10<br>☐☐<br><br>____________<br>(Name of Provider or Facility)	S6.25<br>☐☐<br><br>____________<br>(Name of Provider or Facility)
After (deciding to seek care / being referred), how much time passed before going to the <FIRST/LAST HEALTH PROVIDER>?<br><br>*[Discuss that this might include the time needed to arrange for transportation and money to go to the provider/facility, or to provide home care or go to a traditional provider before going to the health provider.]*<br><br>*[If the child died at home, record the time from decision/referral to death.]*<br><br>*[Mark days, hours &/or minutes as needed: e.g. 01 day, 05 hours, 30 minutes]*		S6.11<br>___ ___ Days<br>*(DK = 99)*<br><br>___ ___ Hours<br>*(DK = 99)*<br><br>___ ___ Minutes<br>*(DK = 99)*	S6.26<br>___ ___ Days<br>*(DK = 99)*<br><br>___ ___ Hours<br>*(DK = 99)*<br><br>___ ___ Minutes<br>*(DK = 99)*
Was there any cost to travel to the <FIRST/LAST HEALTH PROVIDER> or pay for the child's care there?	1. Yes<br>2. No<br>9. Don't know	S6.12<br>☐ ***2 or 9 → SQ6.13***	S6.27<br>☐ ***2 or 9 → SQ6.28***
How did you arrange for the money for these expenses?<br><br>*[Multiple answers allowed.]*	1. Had available ....<br>2. Borrowed ....<br>3. Sold assets ....<br>4. Help from kin/relatives ....<br>5. Community fund ....<br>6. Govt. scheme ....<br>7. Other ....<br>9. Don't know	S6.12.1<br>1. ☐<br>2. ☐<br>3. ☐<br>4. ☐<br>5. ☐<br>6. ☐<br>7. ☐<br>9. ☐	S6.27.1<br>1. ☐<br>2. ☐<br>3. ☐<br>4. ☐<br>5. ☐<br>6. ☐<br>7. ☐<br>9. ☐
What transportation method was used to go there?<br><br>*[Multiple answers allowed.]*	1. Walk ....<br>2. Bicycle/animal/cart/ boat ....<br>3. Bus ....<br>4. Taxi/auto/trecker/motorcycle ....<br>5. Ambulance ....<br>6. Other ....<br>7. Could not arrange transport ....<br>9. Don't know	S6.13<br>1. ☐ ***If only walk***<br>2. ☐ ***→ SQ6.14.1***<br>3. ☐<br>4. ☐<br>5. ☐<br>6. ☐<br>7. ☐ ***→ SQ6.14.1***<br>9. ☐	S6.28<br>1. ☐ ***If only walk***<br>2. ☐ ***→ SQ6.29.1***<br>3. ☐<br>4. ☐<br>5. ☐<br>6. ☐<br>7. ☐ ***→ SQ6.29.1***<br>9. ☐
How much did the transportation cost?		S6.14<br>___ ___ ___ ___ unit<br>*(DK=9999)*	S6.29<br>___ ___ ___ ___ unit<br>*(DK = 9999)*

Did the child reach the <FIRST/LAST HEALTH PROVIDER> before s/he died?<br><br>*[If "No," discuss with respondent to determine correct response: 2, 3 or 4.]*	1. Yes, reached before child died<br>2. No, died before setting out<br>3. No, died on route to this provider<br>4. No, could not reach this provider – did not set out/returned home/took other action<br>9. Don't know	S6.14.1<br>☐ ***2, 3 → SQ6.39***<br>***4, 9 → Inst_4***	S6.29.1<br>☐ ***2-9 → SQ6.39***
How long did it take to travel to the <FIRST/LAST HEALTH PROVIDER>?<br><br>*[Mark hours &/or minutes as needed: e.g. 02 hours, 10 minutes]*		S6.15<br>___ ___ Hours<br>*(DK = 99)*<br><br>___ ___ Minutes<br>*(DK = 99)*	S6.30<br>___ ___ Hours<br>*(DK = 99)*<br><br>___ ___ Minutes<br>*(DK = 99)*
What did the <FIRST/LAST HEALTH PROVIDER> do for <NAME>'s problem?<br><br>*Prompt:* Was there anything else?<br><br>*[Multiple answers allowed.]*	1. Gave oxygen................................<br>2. Helped breathe with bag or mask..<br>3. Gave fluids by mouth ....<br>4. Gave antibiotics by mouth............<br>5. Gave antimalarial by mouth...........<br>6. Gave ORS...................................<br>7. Gave Vitamin A ....<br>8. Gave other medicine by mouth .....<br>9. Gave IM medicine ....<br>10.Gave IV fluids or medicine ....<br>11.Advised to buy outside medicine...<br>12.Did an operation *(specify)* ....<br><br>13.Admitted to hospital......................<br><br>14.Other *(specify)*..............................<br>15.Nothing...<br>99.Don't know	S6.16<br>1. ☐<br>2. ☐<br>3. ☐<br>4. ☐<br>5. ☐<br>6. ☐<br>7. ☐<br>8. ☐ ______________<br>9. ☐<br>10. ☐<br>11. ☐<br>12. ☐ ______________<br><br>13. ☐ stayed __ __ days<br><br>14. ☐ ______________<br>15. ☐ → ***SQ6.18***<br>99. ☐ → ***SQ6.18***	S6.31<br>1. ☐<br>2. ☐<br>3. ☐<br>4. ☐<br>5. ☐<br>6. ☐<br>7. ☐<br>8. ☐ ______________<br>9. ☐<br>10. ☐<br>11. ☐<br>12. ☐ ______________<br><br>13. ☐ stayed __ __ days<br><br>14. ☐ ______________<br>15. ☐ → ***SQ6.33***<br>99. ☐ → ***SQ6.33***
How much did you pay for these treatments and other costs related to the health care, including the admission fee, consultation, lab tests, equipment, and room and food for companions?		S6.17<br>___ ___ ___ ___ ___ unit<br>*(DK = 99999)*	S6.32<br>___ ___ ___ ___ ___ unit<br>*(DK = 99999)*
Did the <FIRST/LAST HEALTH PROVIDER> refer <NAME> to another health provider or facility?	1. Yes<br>2. No<br>9. Don't know	S6.18<br>☐ ***2 or 9 → SQ6.19***	S6.33<br>☐ ***2 or 9 → SQ6.34***
Why was <NAME> referred?<br><br>*[Multiple answers allowed.]*	1. The provider was not capable of managing the problem.................<br>2. Required supplies (e.g., drugs, IV, oxygen) not available...................<br>3. Required equipment (e.g., xray machine) not available.................<br>9. Don't know	S6.18.1<br>1. ☐<br>2. ☐<br>3. ☐<br>9. ☐	S6.33.1<br>1. ☐<br>2. ☐<br>3. ☐<br>9. ☐
Did <NAME> leave the <FIRST/LAST HEALTH PROVIDER> alive?	1. Yes, left alive<br>2. No, died at this provider	S6.19<br>☐ ***2 → VQ5.4***	S6.34<br>☐ ***2 → VQ5.4***
Did the <FIRST/LAST HEALTH PROVIDER> suggest that you do anything for <NAME>'s illness after leaving?	1. Yes<br>2. No<br>9. Don't know	S6.20<br>☐ ***2 or 9 → SQ6.22***	S6.35<br>☐ ***2 or 9 → SQ6.37***

What did the <FIRST/LAST HEALTH PROVIDER> suggest that you do?<br><br>*Prompt:* Was there anything else?<br><br>*[Multiple answers allowed.]*	1. Increase breastfeeding<br>2. Give extra fluids<br>3. Continue feeding<br>4. Give ORS<br>5. Give antibiotic by mouth<br>6. Give antimalarial by mouth<br>7. Give vitamin A by mouth<br>8. Return for follow-up visit<br>9. Return or referred if worse<br>10. Complete the present referral<br>11. Other *(specify)*<br>99. Don't know	S6.20.1<br>1. ☐<br>2. ☐<br>3. ☐<br>4. ☐<br>5. ☐<br>6. ☐<br>7. ☐<br>8. ☐<br>9. ☐<br>10. ☐<br>11. ☐ ________<br>99. ☐ → ***SQ6.22***	S6.35.1<br>1. ☐<br>2. ☐<br>3. ☐<br>4. ☐<br>5. ☐<br>6. ☐<br>7. ☐<br>8. ☐<br>9. ☐<br>10. ☐<br>11. ☐ ________<br>99. ☐ → ***SQ6.37***
Were you able to follow <u>all</u> the advice?	1. Yes<br>2. No<br>9. Don't know	S6.21<br>☐ ***9 → SQ6.22***	S6.36<br>☐ ***9 → SQ6.37***
If <u>not</u> able to follow <u>all</u> the advice, ask: Did you have any concerns or problems that kept you from following the advice?<br><br>*If <u>able</u> to follow <u>all</u> the advice, ask:* Did you have to overcome any concerns or problems to follow the advice?	1. Yes<br>2. No<br>9. Don't know	S6.21.1<br>☐ ***2 or 9 → SQ6.22***	S6.36.1<br>☐ ***2 or 9 → SQ6.37***
What concerns or problems did you have?<br><br>*Prompt:* Was there anything else?<br><br>*[Multiple answers allowed.]*	1. Did not understand instructions<br>2. Too much time from regular duties<br>3. Someone else *(specify)* decided<br>4. Cost too much<br>5. Problem required traditional care<br>6. Thought adivised care not needed<br>7. Thought care might harm the child<br>8. Thought child will die despite care<br>9. No time before go to next provider<br>10. The child died too soon<br>11. Other *(specify)*<br>99. Don't know	S6.21.2<br>1. ☐<br>2. ☐<br>3. ☐ ________<br>4. ☐<br>5. ☐<br>6. ☐<br>7. ☐<br>8. ☐<br>9. ☐<br>10. ☐<br>11. ☐ ________<br>99. ☐	S6.36.2<br>1. ☐<br>2. ☐<br>3. ☐ ________<br>4. ☐<br>5. ☐<br>6. ☐<br>7. ☐<br>8. ☐<br>9. ☐<br>10. ☐<br>11. ☐ ________<br>99. ☐
At the time of leaving the (<FIRST HEALTH PROVIDER> / <LAST HEALTH PROVIDER>), was <NAME>…<br><br>*[Read the choices for each condition.]*	1. Feeding normally, poorly, or not at all<br>2. Alert, drowsy, or unconscious<br>3. Normally active, less active than normal, or not moving	S6.22<br><u>Nrml</u> <u>Med</u> <u>Abnrm</u> <u>DK</u><br>1. ☐ 2. ☐ 3. ☐ 9. ☐<br>1. ☐ 2. ☐ 3. ☐ 9. ☐<br>1. ☐ 2. ☐ 3. ☐ 9. ☐	S6.37<br><u>Nrml</u> <u>Med</u> <u>Abnrm</u> <u>DK</u><br>1. ☐ 2. ☐ 3. ☐ 9. ☐<br>1. ☐ 2. ☐ 3. ☐ 9. ☐<br>1. ☐ 2. ☐ 3. ☐ 9. ☐
If <u>not taken</u> to another health provider, ask: Did you have any concerns or problems that kept you from taking <NAME> to another health provider?<br><br>*If <u>taken</u> to another health provider, ask:* Did you have to overcome any concerns or problems to take <NAME> to another health provider?	1. Yes<br>2. No<br>9. Don't know	S6.23<br>☐ ***2 or 9 → Inst_4***	S6.38<br>☐ ***2 or 9 → SQ6.39***

What concerns or problems did you have?<br><br>*Prompt:* Was there anything else?<br><br>*[Multiple answers allowed.]*	1. Thought no more care needed ....<br>2. No one available to go with her ......<br>3. Too much time from regular duties.<br>4. Someone else *(specify)* decided ....<br>5. Too far to travel ....<br>6. No transportation available............<br>7. Cost (transport, health care, other).<br>8. Not satisfied with available care .....<br>9. Problem required traditional care ...<br>10. Thought child too sick to travel ....<br>11. Thought child will die despite care<br>12. Was late at night ....<br>13. The child died before going..........<br>14. Other *(specify)* ....<br>99.Don't know	S6.23.1<br>1. ☐<br>2. ☐<br>3. ☐<br>4. ☐ ______________<br>5. ☐<br>6. ☐<br>7. ☐<br>8. ☐<br>9. ☐<br>10. ☐<br>11. ☐<br>12. ☐<br>13. ☐ → ***SQ6.39***<br>14. ☐ ______________<br>99. ☐	S6.38.1<br>1. ☐<br>2. ☐<br>3. ☐<br>4. ☐ ______________<br>5. ☐<br>6. ☐<br>7. ☐<br>8. ☐<br>9. ☐<br>10. ☐<br>11. ☐<br>12. ☐<br>13. ☐<br>14. ☐ ______________<br>99. ☐
Inst_4: Check SQ6.5 → If taken to another health provider…		***…go to SQ6.24 (LAST PROVIDER)***	

S6.39	How many days after (first noticing the illness / <LAST ACTION SQ6.5> / leaving the first/last health provider) did <NAME> die?<br><br>*[If SQ6.4 = 2 (No care given), then read: "…first noticing the illness…"]*	___ ___ Days<br>*(<1 = 00; DK = 99)*

Inst_5: If SQ6.4 = 2 (No care given) or
if SQ6.5 ≠ "Health Provider" (Never took and never tried to take to a health provider) → VQ5.10

VA Section 5: Health records (FOR STILLBIRTHS, NEONATAL & CHILD DEATHS 0—59 MONTHS OLD)

V5.4	Do you have any health records that belonged to the deceased?	1. Yes<br>2. No<br>9. Don't know	☐ ***2 or 9 → VQ5.10***
V5.5	Can I see the health records?	1. Yes<br>2. No	☐ ***2 → VQ5.10***
V5.6	*Record the dates of the two most recent visits*		___ ___/___ ___/___ ___ ___ ___<br>D D M M Y Y Y Y<br>*(DK = 99/99/9999)*<br><br>___ ___/___ ___/___ ___ ___ ___<br>D D M M Y Y Y Y<br>*(DK = 99/99/9999)*
V5.7	*Record the two most recent weights on those dates*		___ ___ ___ ___ Grams<br>*(DK = 9999)*<br><br>___ ___ ___ ___ Grams<br>*(DK = 9999)*
V5.8	*Record the date of the last note*		___ ___/___ ___/___ ___ ___ ___<br>D D M M Y Y Y Y<br>*(DK = 99/99/9999)*

V5.9	*Transcribe the note*		
	____________________<br>____________________<br>____________________<br>____________________<br>____________________<br>____________________<br>____________________		
V5.10	Was a death certificate issued?	1. Yes<br>2. No<br>9. Don't know	☐ ***2 or 9 → SQ1.1***
V5.11	Can I see the death certificate?	1. Yes<br>2. No	☐ ***2 → SQ1.1***
V5.12	*Record the immediate cause of death from the death certificate*		
V5.13	*Record the first underlying cause of death from the death certificate*		
V5.14	*Record the second underlying cause of death from the death certificate*		
V5.15	*Record the third underlying cause of death from the death certificate*		
V5.16	*Record the contributing cause of death from the death certificate*		

SA Module 1: The mother and her household (FOR STILLBIRTHS, NN & CHILD DEATHS 0—59 MONTHS OLD)

Read: Now I would like to ask you some other questions about (yourself / the child's mother).

[Read "…the child's mother." If the respondent is not the mother.

Inst_1: If GQ4.3 = 1 (Respondent is the mother) → SQ1.4

S1.2	How old (is the child's mother / was the child's mother when she died)?<br><br>*[Read "…was the child's mother…" if she died.]*		___ ___ Years<br>*(DK = 99)*
S1.3	How many years of school did the mother complete?		___ ___ Years<br>*(<1 = 00; DK = 99)*
S1.4	(Are you / Is/Was the child's mother)…<br><br>*[Read "…Is/Was the child's mother…" if the respondent is not the mother.]*<br><br>*[Read the choices to the respondent.]*	1. Married?<br>2. Living with a man?<br>3. Widowed?<br>4. Divorced, separated, or deserted?<br>5. Single (never married/lived w/a man)?<br>9. Don't know	☐ ***5 or 9 → Inst_2***
S1.4.1	How old (were you when you / was she when she) first married (or lived with a man)?<br><br>*[Read "…was she when she…" if the respondent is not the mother.]*<br>*[Read "…married or lived with a man?" if SQ1.4 = "2. Living with a man"]*		___ ___ Years<br>*(DK = 99)*

S1.4.2	How many years of school did (your / her) (husband / partner) complete?<br><br>*[Read "…her…" if the respondent is not the mother.]*<br>*[Read "…partner…" if she is living with a man.]*		___ ___ Years<br>*(<1 = 00; DK = 99)*
Inst_2: *Read:* Now I would like to ask you some questions about (your / the mother's) household. Please remember that all information will be kept confidential.<br><br>*[SBs & NN deaths: If the respondent is not the mother, read "…the mother's…;" and ask SQ1.5–1.11 about the mother's household.*<br><br>*Older deaths: Always read "…your…;" and ask SQ1.5–1.11 about the respondent's household.]*			
S1.5	Who was the main breadwinner of (your / the mother's) family during the (last days of the pregnancy / child's fatal illness)<br><br>*[SBs/NN deaths: Read "…last days…"; Older deaths: Read "…child's…"]*	1. Child's father<br>2. Child's mother<br>3. Other<br>9. Don't know	☐ ***9 → SQ1.7***
S1.6	At that time, what kind of work did the main breadwinner mostly do?	1. Farmer/agricultural worker<br>2. Poultry or cattle raising<br>3. Domestic servant<br>4. Home-based manufacturing<br>5. Unskilled laborer<br>6. Semi-skilled laborer/service provider<br>7. Factory worker, blue collar service<br>8. Business owner<br>9. Professional/technician<br>10. Other *(specify)* ....<br>11. Overseas worker<br>99. Don't know	☐☐<br><br>____________________________
S1.7	Is this the house (where we are now) where (you / the mother) stayed during the (last days of the pregnancy / child's fatal illness)?<br><br>*[SBs/NN deaths: Read "…last days…" Older deaths: Read "…child's…"]*<br><br>*[Read "…where we are now…" if needed to clarify which house you are talking about.]*	1. Yes<br>2. No<br>9. Don't know	☐ ***1 → SQ1.10***<br>***9 → VQ5.17***
S1.8	Where did (you / the mother) stay at that time?<br><br>*Probe:* Where did (you / the mother) stay during the illness events?<br><br>*[Mark "1" only if her usual residence was not her in-laws or other relatives.]*	1. Her own home at that time (other than with her in-laws) *(Interviewer: Use this code just if she moved after the death.)*<br>2. Her in-law's home<br>3. Her parent's home<br>4. Her brother's home<br>5. Other *(specify)* ....<br>9. Don't know	☐ ***9 → VQ5.17***<br><br>____________________________
S1.9	What is the address of the place where (you / she) stayed?	State ______________________________<br><br>LGA ________________________________	☐☐☐<br>☐☐☐
S1.10	At the time of the illness events, how long had (you / the mother / your <RELATIVES> / the mother's <RELATIVES>) been living continuously in (this / that) community?<br><br>*[Read "…<RELATIVES…" if SQ1.8 = 2-5 (s/he stayed with her/his relatives)].*		___ ___ Years<br>*(<1 = 00; DK =99)*
S1.11	How long does it take to reach the health provider or facility where (you / the mother) normally (go(es) / went) from (this / that) place?		___ ___ Hours<br>*(DK = 99)*

	[Mark hours &/or minutes as needed: e.g. 01 hour, 30 minutes]		___ ___ Minutes<br>*(DK = 99)*
Inst_3 → SQ2.1.1 (if including optional Module 2) or VQ5.17			
**SA Module 2: Social capital (OPTIONAL MODULE—FOR SBs, NN & CHILD DEATHS 0–59 MONTHS OLD)**<br><br>*Read:* Now, I have some questions about (your / the mother's / your <RELATIVES'> / the mother's <RELATIVES'>) community.<br><br>*[SBs and NN deaths: If the respondent is not the mother, read "…the mother's…" or "…the mothers' <RELATIVES'>...;" and ask SQ2.1.1–SQ2.3.1 about the mother and her community or her relatives' community.*<br><br>*Older deaths: Always read "…your…" or "…your <RELATIVES'>…;" and ask SQ2.1.1–SQ2.3.1 about the respondent and her/his community or her/his relatives' community.*<br><br>*All deaths: Ask about the relatives' community if s/he stayed with her/his relatives during the illness events.]*			
S2.1.1	In the last 3 years, did the people in the (village / neighborhood) work together on any of the following issues that affect the entire community or part of the community?<br><br>*Read all the issues and mark ("X") Yes, No or DK for each one; then enter the code.]*	1. Education/schools<br>2. Health services/clinics<br>3. Paid job opportunities<br>4. Credit/finance<br>5. Roads<br>6. Public transportation<br>7. Water distribution<br>8. Sanitation services<br>9. Agriculture<br>10. Justice/conflict resolution<br>11. Security/police services<br>12. Mosque/church/temple<br>13. Other<br><br>*(specify)*	Yes No DK<br>1. ☐ 2. ☐ 9. ☐<br>1. ☐ 2. ☐ 9. ☐<br>1. ☐ 2. ☐ 9. ☐<br>1. ☐ 2. ☐ 9. ☐<br>1. ☐ 2. ☐ 9. ☐<br>1. ☐ 2. ☐ 9. ☐<br>1. ☐ 2. ☐ 9. ☐<br>1. ☐ 2. ☐ 9. ☐<br>1. ☐ 2. ☐ 9. ☐<br>1. ☐ 2. ☐ 9. ☐<br>1. ☐ 2. ☐ 9. ☐<br>1. ☐ 2. ☐ 9. ☐<br>1. ☐ 2. ☐ 9. ☐<br><br>______________________
		Code:<br>1. One or more issues identified<br>2. No issue identified	☐
S2.2	(Were you / Was the mother) able to turn to any persons, groups or organizations in the community for help during (the pregnancy / (or) the child's fatal illness)?<br><br>*[Read "…the pregnancy?" for SBs; or "…the pregnancy or the child's fatal illness?" for NN deaths; or "…the child's fatal illness for older deaths.]*	1. Yes<br>2. No<br>9. Don't know	☐ ***2 or 9 → SQ2.3.1***
S2.2.1	Did (you / she) turn to any of the following for help?<br><br>*[Read all the options and mark ("X") Yes, No or DK for each; then enter the code.]*	1. Family<br>2. Neighbors<br>3. Friends<br>4. Religious leader or group<br>5. Community leader<br>6. Police<br>7. Patron/employer/benefactor<br>8. Political leader<br>9. Mutual support group s/he belongs to<br>10. Assistance organization to which s/he does not belong<br>11. Other<br><br>*(specify)*	Yes No DK<br>1. ☐ 2. ☐ 9. ☐<br>1. ☐ 2. ☐ 9. ☐<br>1. ☐ 2. ☐ 9. ☐<br>1. ☐ 2. ☐ 9. ☐<br>1. ☐ 2. ☐ 9. ☐<br>1. ☐ 2. ☐ 9. ☐<br>1. ☐ 2. ☐ 9. ☐<br>1. ☐ 2. ☐ 9. ☐<br>1. ☐ 2. ☐ 9. ☐<br>1. ☐ 2. ☐ 9. ☐<br>1. ☐ 2. ☐ 9. ☐<br>1. ☐ 2. ☐ 9. ☐<br><br>______________________

		Code:<br>1. One person/group identified<br>2. Two or more persons/groups identified<br>3. No person/group identified	☐ ***3 → SQ2.3.1***
S2.2.2	(Is this / Are these) the same person(s) or group(s) (you / she) would usually turn to for help with a serious problem?	1. Yes<br>2. No<br>9. Don't know	☐
S2.3.1	(Have you or your / Has the mother or her) family ever been denied any of the following community services?<br><br>*Read all the options and mark ("X") Yes, No or DK for each; then enter the code.]*	1. Education/schools<br>2. Health services/clinics<br>3. Paid job opportunities<br>4. Credit/finance<br>5. Transportation<br>6. Water distribution<br>7. Sanitation services<br>8. Agricultural extension<br>9. Justice/conflict resolution<br>10. Security/police services<br>11. Other<br><br>*(specify)*	Yes No DK<br>1. ☐ 2. ☐ 9. ☐<br>1. ☐ 2. ☐ 9. ☐<br>1. ☐ 2. ☐ 9. ☐<br>1. ☐ 2. ☐ 9. ☐<br>1. ☐ 2. ☐ 9. ☐<br>1. ☐ 2. ☐ 9. ☐<br>1. ☐ 2. ☐ 9. ☐<br>1. ☐ 2. ☐ 9. ☐<br>1. ☐ 2. ☐ 9. ☐<br>1. ☐ 2. ☐ 9. ☐<br>1. ☐ 2. ☐ 9. ☐<br><br>__________________
		Code:<br>1. One service denied<br>2. Two or more services denied<br>3. No denied service identified	☐
V5.165	*Read:* Now I have four last questions about the child and the child's mother.<br><br>Before the fatal illness began, did the child suffer from HIV/AIDS?	1. Yes<br>2. No<br>8. Refused to answer<br>9. Don't know	☐
V5.17	(Have you / Has the deceased's biological mother) ever been tested for "HIV"?	1. Yes<br>2. No<br>8. Refused to answer<br>9. Don't know	☐ ***2-9 → VQ5.19***
V5.18	Was the "HIV" test ever positive?	1. Yes<br>2. No<br>8. Refused to answer<br>9. Don't know	☐
V5.19	(Have you / Has the deceased's biological mother) ever been told she had "AIDS" by a health worker?	1. Yes<br>2. No<br>8. Refused to answer<br>9. Don't know	☐

VA Section 6 & SA Module 7: Open ended response & interviewer comments/observations (FOR ALL DEATHS)

Read: Thank you for answering the many questions that I've asked. Would you like to tell me about <NAME>'s illness in your own words? Also, is there anything else about her/his illness that I did not ask and you would like to tell me about?

After the respondent(s) finishes, ask: Is there anything else?

Write the respondent's exact words. After s/he has finished, read this back and ask her to correct any errors in what you wrote.

____

____

____

____

____

____

____

____

____

____

____

____

____

____

END OF INTERVIEW
THANK RESPONDENT FOR HER/HIS PARTICIPATION

Interviewer: Use this space to write down your comments and observations about the interview.

____

____

____

____

____

____

____
