# Supplementary figures and images for: Beyond causes of death: The social determinants of mortality among children aged 1-59 months in Nigeria from 2009 to 2013
Source: PLoS One. 2017 May 31;12(5):e0177025. doi: 10.1371/journal.pone.0177025 (PMC5451019; doi:10.1371/journal.pone.0177025)

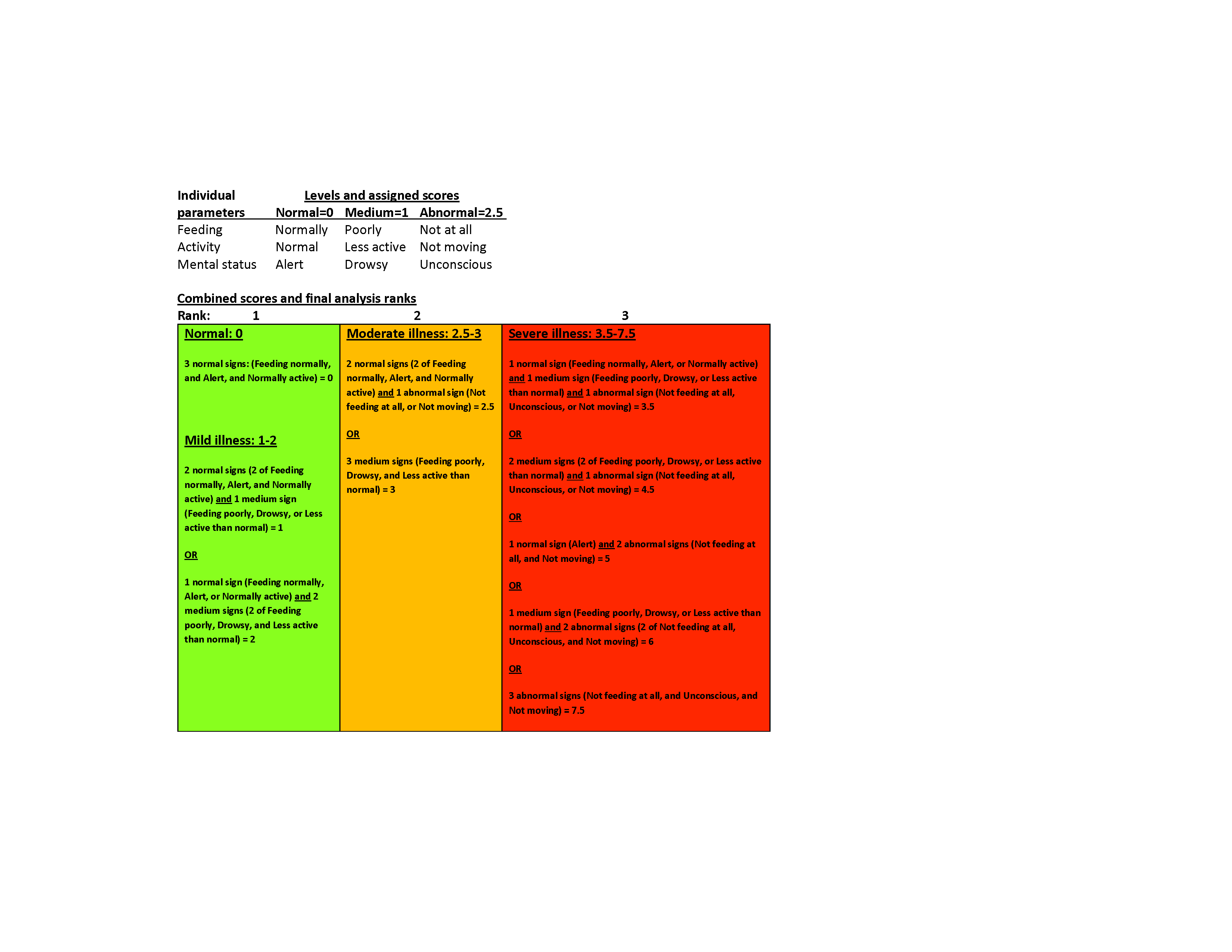

Supplement: S1 Fig — (TIFF) [file pone.0177025.s002.tiff]
